# A Bioengineered Stable Protein 1‐Hemin Complex with Enhanced Peroxidase‐Like Catalytic Properties

**DOI:** 10.1002/smsc.202400025

**Published:** 2024-03-19

**Authors:** Yara Zeibaq, Oren Bachar, Jenia Sklyar, Noam Adir, Omer Yehezkeli

**Affiliations:** ^1^ Department of Biotechnology and Food Engineering Technion—Israel Institute of Technology Haifa 3200003 Israel; ^2^ Schulich Faculty of Chemistry Technion—Israel Institute of Technology Haifa 320000 Israel; ^3^ Russell Berrie Nanotechnology Institute Technion—Israel Institute of Technology Haifa 3200003 Israel; ^4^ The Nancy and Stephen Grand Technion Energy Program Technion—Israel Institute of Technology Haifa 3200003 Israel

**Keywords:** artificial enzyme, biocatalysis, glucose biosensing, peroxidase, stable protein 1

## Abstract

Enzymes have gained their unique efficiency and catalytic activity through billions of years of evolution, perfecting their active site to a desired reaction. Inspired by nature, a novel enzyme‐mimicking platform is designed based on stable protein 1 (SP1) to create a nano‐compartment that mimics peroxidase activity. The biohybrid reveals enhanced activity over the hemin cofactor alone and improved stability in organic solvents in comparison to native peroxidase. Furthermore, the utilization of the obtained biohybrid in an optical glucose biosensing platform is shown. The biohybrid crystallographic structure is solved, indicating that the SP1 structure is not affected by the hemin coordination. This work opens the path for developing new cofactor binding centers in engineered protein scaffolds for various artificial catalytic processes.

## Graphical abstract

Peroxidase is a heme‐containing enzyme that has been broadly used in medical diagnosis, environmental treatments, and chemical syntheses. Its main limitation is low expression yields and poor stability in harsh environments. Herein, the feasibility and the use of Stable protein 1, a thermo‐stable and stress‐responsive protein, conjugated with hemin as a nano‐compartment for peroxidase mimicry is shown.

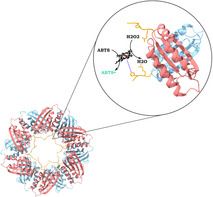

## 1 Introduction

Enzymes are natural catalysts that drive biological and chemical reactions with exceptional efficiencies.^[^ ^1^ ^]^ Their high substrate selectivity provides a route for pure product production at high rates.^[^ ^2^ ^]^ The enzymes’ unique capabilities and properties make them attractive for many industrial processes, such as in the food and pharmaceutical industries. Furthermore, enzymes greatly contribute to the production of renewable energy such as biodiesel.^[^ ^3^ ^]^ During the last decades, biotechnology processes have greatly evolved, enabling routine mass production of enzymes in bacteria, regardless of their original biological source. Despite their exceeding potential, in many cases, the use of enzymes is limited due to low expression yields, poor solubility, and poor stability under harsh environments. Furthermore, enzyme production is usually achieved with expensive, complex, and polluting purification processes.^[^ ^4^, ^5^, ^6^ ^]^ As a result of the aforementioned, the commercial utilization of enzymes has not yet reached its full potential.^[^ ^7^, ^8^ ^]^ These limitations are in many cases a consequence of the differences between the enzymes’ natural environment and the required industrial conditions.^[^ ^8^ ^]^ To overcome these issues, reengineering, and enzymes reconstruction have been performed to allow optimization and adaptation to the industrial requirements. These bioengineering efforts include biological catalysts, biomimicry configurations, and de novo enzymes.^[^ ^9^, ^10^ ^]^ For example, nanozymes, which mimic the enzymes’ natural active site environment have been designed and tested.^[^ ^11^, ^12^ ^]^ Recently, Ding et al. developed a 1D nanowire with Fe–N_4_ sites doped with phosphorous to enhance its peroxidase‐like activity for biosensing assays.^[^ ^13^ ^]^ Deng et al. developed an artificial metalloenzyme by substituting myoglobin hemin with cobalt porphyrin for photocatalytic CO_2_ reduction.^[^ ^14^ ^]^ Using different approaches, Arnold and co‐workers have developed directed evolution tools that extend the known natural reactions of enzymes to new ones.^[^ ^15^, ^16^, ^17^ ^]^ Furthermore, rational design methodologies were developed using both computational and experimental tools to perfect novel reactions.^[^ ^9^, ^18^, ^19^ ^]^ Tezcan and coworkers,^[^ ^20^ ^]^ have introduced an approach to construct new protein interfaces by exploiting metal coordination.

Horseradish peroxidase (HRP) is a ubiquitous heme‐containing enzyme purified from the roots of *Armoracia rusticana.* The HRP enzyme facilitates the oxidation of aromatic molecules while reducing hydrogen peroxide. Furthermore, it plays a major role in the humification process, and in addition, it is involved in xenobiotic metabolism.^[^ ^21^, ^22^ ^]^ HRP has been broadly used in medical diagnosis, environmental treatments, and chemical synthesis.^[^ ^23^, ^24^, ^25^, ^26^, ^27^, ^28^ ^]^ Recently, approaches to mimic the enzyme's active site have been developed.^[^ ^29^ ^]^ These include, hemin‐free fibrils,^[^ ^30^ ^]^ coordinated hemin to organic or inorganic scaffolds,^[^ ^31^, ^32^ ^]^ DNA G‐quadruplex formation,^[^ ^33^, ^34^, ^35^, ^36^, ^37^ ^]^ proteins,^[^ ^38^, ^39^, ^40^, ^41^, ^42^ ^]^ supramolecular and nano hydrogels,^[^ ^43^, ^44^, ^45^ ^]^ copolymers,^[^ ^46^, ^47^ ^]^ carbon nanotubes,^[^ ^48^, ^49^ ^]^ and hybrids of the above.^[^ ^50^, ^51^ ^]^ However, the construction of a flexible active microenvironment while maintaining high stability under harsh conditions, remains a challenge. Watkins et al.^[^ ^52^ ^]^ have demonstrated the power of the maquette approach, where non‐natural four‐α‐helix bundles are used for the construction of thermostable de novo enzyme. The enzyme kinetics was similar to or surpassed some of the natural peroxidases. In addition, it retained its activity in organic solvents.^[^ ^52^ ^]^

Stable protein 1 (SP1), isolated from *Populus tremula*, is a homo dodecamer protein with superior thermo‐stability.^[^ ^53^ ^]^ Its monomers self‐assemble into a ring‐shaped complex resulting in a central pore with a diameter of 2.5 nm. The 12 monomers are self‐assembled to form a stable “doughnut‐like” structure comprising all of the monomers’ N‐termini facing the protein cavity.^[^ ^53^, ^54^ ^]^ Medalsy et al. investigated the parameters affecting the formation of controlled biohybrid nanostructures based on SP1 units and embedded gold nanoparticles.^[^ ^54^ ^]^ Our research group has recently presented methodologies for biosynthesis size‐controlled palladium NPs in bioengineered SP1 protein. These 2–3 nm sized nanoparticles were grown in the SP1 protein expressed in *E. coli.* The formed biohybrids were further utilized in vivo for the acetylene‐to‐ethylene reduction process.^[^ ^55^ ^]^ In follow‐up work, bioengineered SP1 has been utilized for the biosynthesis of crystalline CdS nanoparticles in a size‐controlled, homogenous, and ordered approach.^[^ ^56^ ^]^ These CdS QDs were then utilized for a light‐driven biotic/abiotic‐based apparatus for the synthesis of chiral (R)‐2‐methyl pyrrolidine.

Herein, we present an alternative mimicry platform for the peroxidase enzyme. The designed biocatalyst is based on a bioengineered SP1 protein scaffold coordinated to a hemin cofactor. The SP1 N‐terminus where bioengineered to display metal binding peptides (MBPs). These MBPs are known for their high affinity to bind metals or semiconductors.^[^ ^57^, ^58^, ^59^, ^60^ ^]^ We hypothesized that coordinating hemin to the SP1's inner cage could simulate an enzyme‐like microenvironment. We show that only specific SP1 variants that display metal binding peptides can enable a peroxidase‐like activity in contrast to the unbonded peptides. We further demonstrate the utilization of the developed “enzyme‐like” scaffold in optical biosensors for glucose and for catalytic activity under the harsh conditions of organic solvents. In addition, the crystal structure of the bioengineered SP1 was determined, showing some unexpected crystal lattice packing architectures that extend the knowledge of previously determined SP1 crystal structures.

## 2 Results and Discussion

**Scheme 1:**
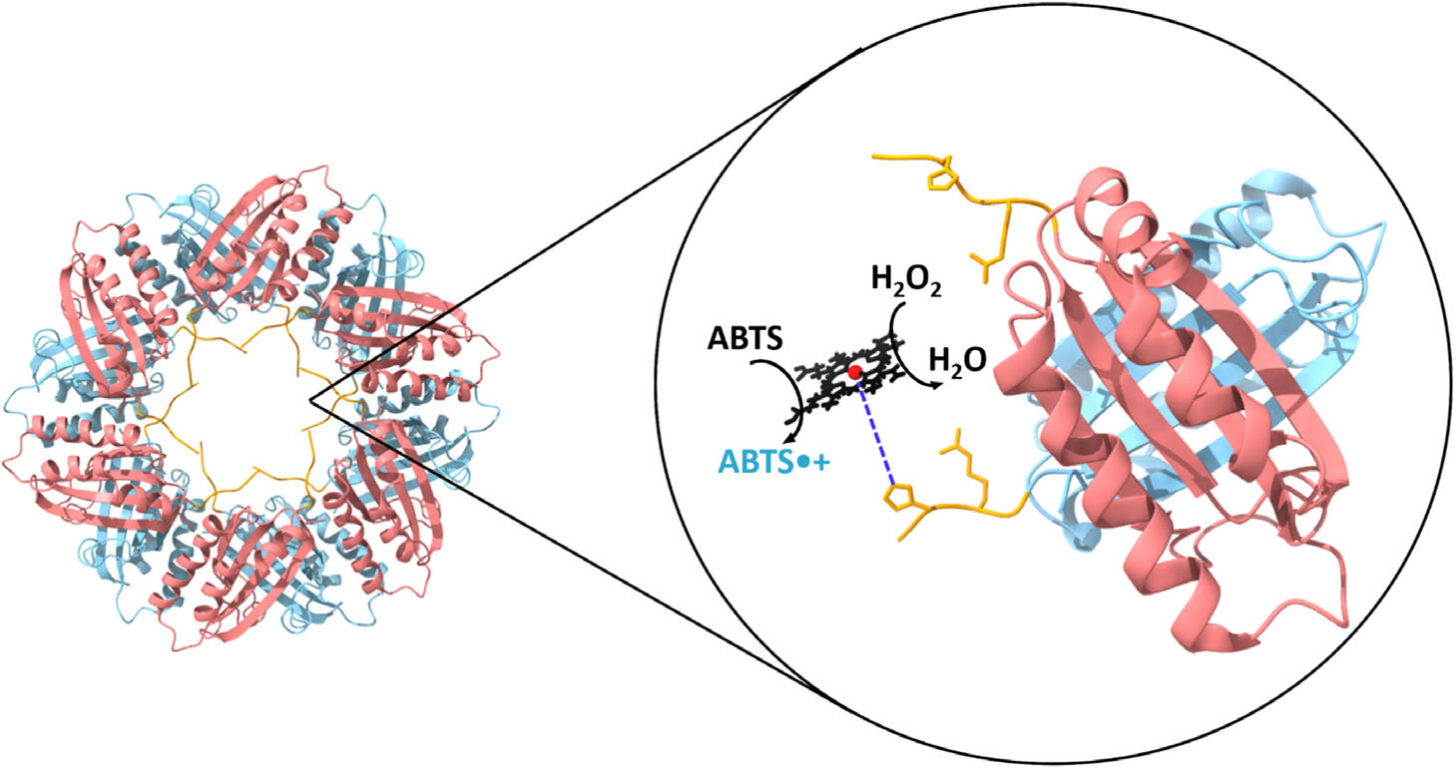
An SP1@hemin complex model based on the 1TR0 structure deposited in the PDB. Left, the dodecameric, double ring SP1crystal structure. Right, the hybrid active site where pink and blue represent two monomers. The N‐termini are extended with the MBPs presented in orange. The hemin‐His coordination bond is shown in a blue dashed line. The figure was prepared using ChimeraX.^[^ ^96^ ^]^

The designed artificial platform consists of a hemin cofactor coordinated with the SP1 protein. To enable complexation, we incorporated metal‐binding peptides into the N‐terminal SP1 encoding sequences. It has been shown that these peptides exhibit high affinity to specific targeted material surfaces. Four SP1 variants were examined, each consisting of a different metal‐binding peptide (MBP) at the N‐terminus, as illustrated in **Scheme** **1**. The bioengineered SP1 variants include coordination positions and structures that resemble the peroxidase enzyme active site. We show that specific amino acid sequences are required for efficient coordination with the hemin molecules. For that, we designed SP1 variants with three different sequences, binding peptide 1 (BP1‐SP1, MMHGKTQATSGTIQS), binding peptide 2 (BP2‐SP1, MGDVHHHGRHGAEHADI, binding peptide 3(BP3‐SP1, MSVTQNKY), and a his‐tagged variant, binding peptide 4 (BP4‐SP1, HHHHHH). All variants were overexpressed in *E. coli* and further purified using anion exchange and size exclusion chromatography (see Materials and methods). The purified variants were then incubated with hemin molecules to examine their ability to coordinate with the Fe center and further screened for active complex formation that exhibits peroxidase‐like activity. It should be noted that the MBP classification is based on previous work that examined their affinity to nanoparticles or material surfaces.^[^ ^54^, ^55^, ^56^, ^61^ ^]^ Here, we examined the ability of these amino acid sequences to coordinate with the hemin cofactor and enable “enzyme‐like” catalytic activity.

**Figure 1:**
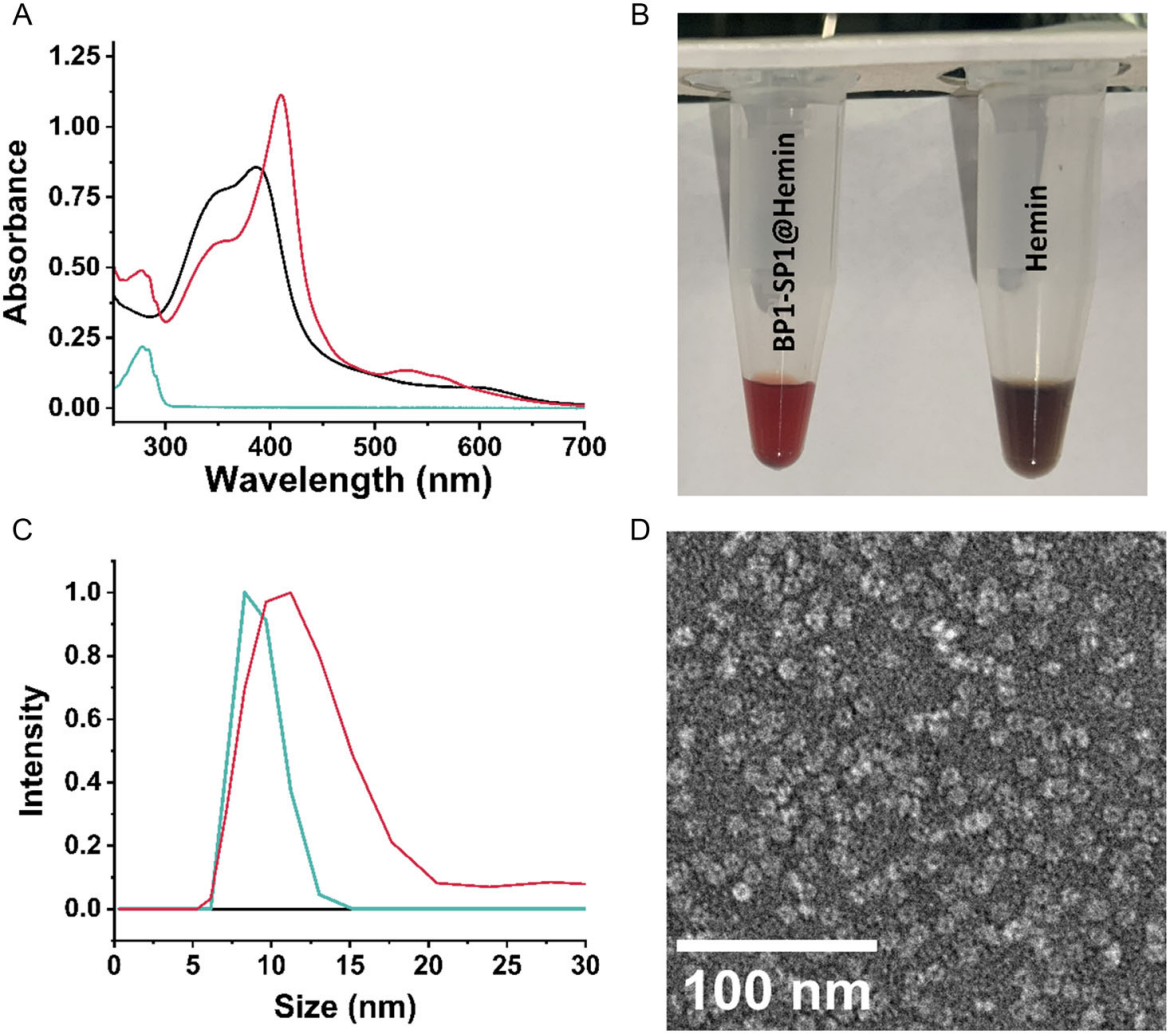
A) Absorption spectra of BP1‐SP1@hemin complex (red), free hemin (black), and free BP1‐SP1 (green) BP1‐SP1 2.5 μm, hemin 20 μm. The biohybrids and hemin solution consists of 95% HEPES and 5% DMSO B) Photograph of the prepared BP1‐SP1@hemin and hemin samples. C) dynamic light scattering of BP1‐SP1@hemin complex (red), free hemin (black), and free BP1‐SP1 (green) BP1‐SP1 1.25 μm, hemin 10 μm dissolved in 97.5% HEPES and 2.5% DMSO. D) Transmission electron microscopy (TEM) image of BP1‐SP1@hemin complex.

The SP1 and hemin molecules interaction was followed using UV and visible light absorption spectroscopy as presented in **Figure** **1**A. As depicted, free hemin absorbs at 390 nm with an additional shoulder at 365 nm, black curve. The shoulder can be attributed to the μ‐oxo bihemin, a dimeric form of hemin,^[^ ^62^ ^]^ while the 390 nm peak is to the monomeric form.^[^ ^62^ ^]^ The peak at 610 nm can be attributed to the Q band value of μ‐oxo bihemin.^[^ ^62^, ^63^ ^]^ When assembled, the BP1‐SP1@hemin complex exhibits a Soret peak at 410 nm, a shoulder at 365 nm, and low‐intensity bands at 530 nm and 568 nm, (Figure 1A, red curve). The observed redshift indicates that complexation occurred, linking the hemin and the BP1‐SP1 through coordination chemistry.^[^ ^64^ ^]^ The appearance of the new peaks indicates the presence of low‐spin hemin.^[^ ^65^, ^66^ ^]^ It should be noted that at high hemin concentrations, the μ‐oxo bihemin absorbance shoulder is visible. However, in diluted solutions, the bihemin dimer is less likely to be formed, and therefore less pronounced in the spectrum.^[^ ^36^ ^]^ The absorbance measurements showed that the BP1‐SP1 protein solely absorbs at 280 nm, (Figure 1A, green curve). The other SP1 constructs were also examined, (Figure S2, Supporting Information). As opposed to the BP1‐SP1, the wild type SP1 lacking a fused peptide, the BP3‐SP1 did not exhibit any absorbance shifts while mixed with hemin. Surprisingly, BP2‐SP1 is unable to bind hemin although it contains multiple histidine residues. This may be due to the creation of an unfavorable arrangement of the N‐terminus limiting hemin binding. The BP4‐SP1@hemin complex exhibited a minor redshift, reaching 403 nm upon the addition of the protein. Also, we could observe the emergence of new peaks at 530 and 568 nm, however with lower intensity compared to the BP1‐SP1@hemin, (Figure S2, Supporting Information). In addition, by comparing the BP1‐SP1@hemin to the free hemin solution we could observe a spectral change. This color change indicated that indeed the complexation occurred. (Figure 1B).

**Figure 2:**
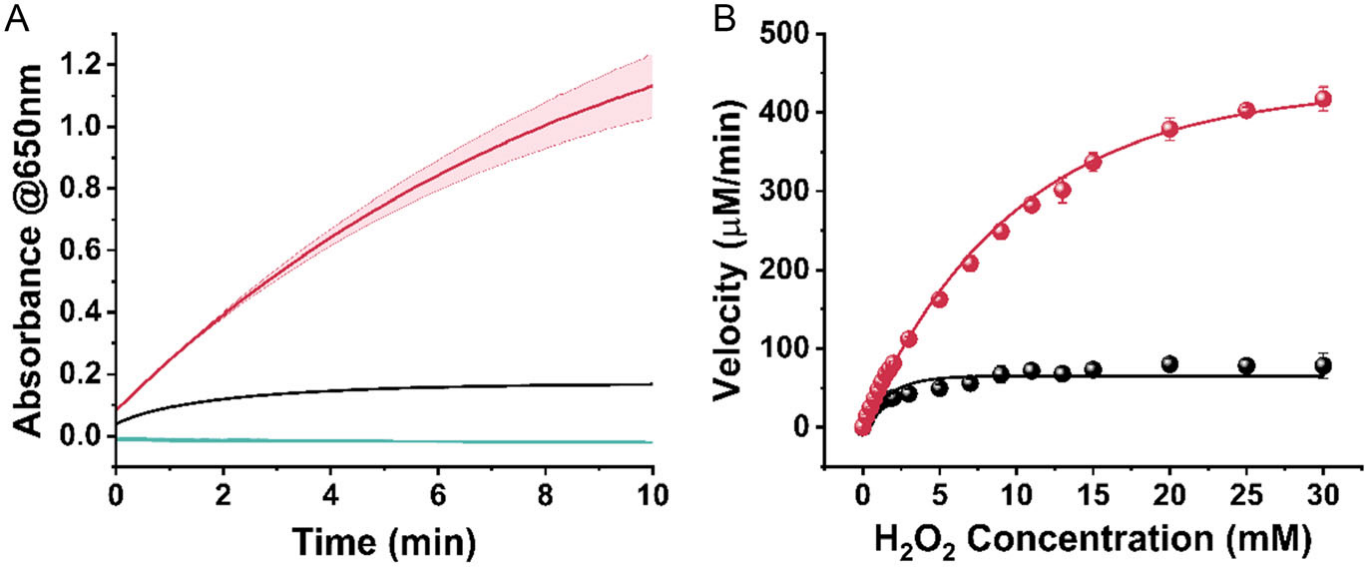
A) The peroxidase‐like activity of BP1‐SP1@hemin complex (red), free hemin (black), and free BP1‐SP1 (green), in the presence of H_2_O_2_ 1.28 mm, ABTS 1.28 mm, BP1‐SP1 0.25 μm, hemin 2 μm, HEPES buffer 10 mm pH = 8, the reaction was initiated by the addition of H_2_O_2_. The absorbance of ABTS was measured at 650 nm for 10 min. The light area around the curve indicates the standard deviation of three independent experiments. B) The Michalis–Menten kinetic curve of BP1‐SP1@hemin complex (red dots) and hemin (black dots), ABTS 10 mm, BP1‐SP1 0.29 μm, hemin 2.3 μm, HEPES buffer 10 mm pH = 8. The reaction was initiated by the addition of H_2_O_2_. The absorbance of ABTS was measured at 650 nm for 1 min. Error bars indicate the standard deviation of independent triplicates.

As noted above, the SP1 protein is self‐assembled into a dodecameric double “doughnut‐like” ring with all of the N‐termini directed into the internal cavity. Therefore, on each side, six expressed peptides can coordinate with single or multiple hemin molecules. To elucidate the ratio between the protein and the hemin molecule, the hemin concentration was fixed while increasing the concentration of the added BP1‐SP1. The BP1‐SP1 addition led to increased absorption at 410 nm which can be attributed to the formation of coordinated hemin monomer with the BP1‐SP1 (Figure S3, Supporting Information). Furthermore, the BP1‐SP1 addition induced a decrease in the 390 nm peak (Figure S3, Supporting Information). This can be ascribed to the reduction of dimeric hemin formation. Moreover, above a 1:8 ratio, the spectrum did not further change, (Figure S3, Supporting Information). These results imply that above the 1:8 ratio, the protein is fully saturated by the hemin molecules. To further verify that indeed the hemin coordinates with the BP1‐SP1 we performed a dynamic light scattering (DLS) analysis. As depicted in Figure 1C, upon complexation with the hemin, the measured BP1‐SP1 dimensions shifted, presenting an average size of 11.23 nm compared to 8.2 nm measured for the unreacted protein, (Figure 1C). Electrochemical methods were also conducted to examine the hemin, BP1‐SP1@hemin, and the BP1@hemin redox potentials and electrocatalytic processes. The oxidation state of the hemin iron center was followed by cyclic voltammetry under anaerobic conditions. An oxidation peak at 0.06 V, which can be attributed to the oxidation of the iron center (Fe^2+^ to Fe^3+^), and a reduction peak at −0.15 V(Fe^3+^ to Fe^2+^) was observed.^[^ ^67^ ^]^ As anticipated, the addition of H_2_O_2_ induced an increase in the cathodic peak, which can be attributed to the electrocatalytic reduction of H_2_O_2_ (Figure S4, Supporting Information). The BP1‐SP1@hemin shows a reduction peak at ‐0.1 V while no clear oxidation peak. (Figure S5, Supporting Information). Similarly to the hemin electrocatalytic activity, the addition of H_2_O_2_ induced electrocatalytic currents. The BP1@hemin samples showed two reduction peaks; one at −0.01 V, and an ambiguous peak at −0.17 V (Figure S6, Supporting Information). The current increased upon H_2_O_2_ addition and the reduction peak was more pronounced. However, the electrocatalytic currents were less pronounced in comparison to the hemin and BP1‐SP1@hemin samples. While all configurations presented electrocatalytic activity in the presence of H_2_O_2_, only the BP1‐SP1@hemin showed onset that can be attributed to the reduction peak. These results may be explained by the electrode passivation occurred by the SP1 protein, where only the active site enables electrocatalytic reduction. This is a common behavior of enzymes on electrode surfaces. To confirm that the BP1‐SP1 maintains its structure in the presence of hemin we imaged the hybrid structures by TEM. For that, the hybrid samples were deposited on the TEM grids, followed by uranyl staining. As depicted in Figure 1D, the BP1‐SP1 maintained its ringed structure, similar to the unreacted BP1‐SP1, (Figure S7, Supporting Information). We then examined the “peroxidase‐like” activity of the BP1‐SP1@hemin hybrids. The catalytic activity of the BP1‐SP1@hemin was investigated using 2,2′‐azino‐bis(3‐ethylbenzothiazoline‐6‐sulfonic) acid (ABTS), a commonly used dye probe for peroxidase activity, (Scheme 1). Upon the addition of BP1‐SP1@hemin and H_2_O_2_ as a substrate, the oxidation of the ABTS could be followed spectroscopically at 650 nm. The resulting catalytic activity was compared to the free hemin activity. As depicted, a six‐fold increase in the catalytic activity was observed, (**Figure** **2**A). We further examined the other SP1 variants. As depicted, except for the BP4‐SP1@hemin hybrid, all of the other variants did not show any enhanced catalytic activity. The BP4‐SP1@hemin presented improved activity, however, it was ≈50% lower than the BP1‐SP1@hemin, (Figure S8, Supporting Information). The improved activity can be attributed to the histidine 3 and arginine 16 positioned in the BP1 sequence as these two amino acids participate in the catalytic reaction in natural peroxidases.^[^ ^48^, ^68^ ^]^ While the Arg exists in both sequences, lower activity was achieved with a His‐tag. We attribute the different catalytic activity to the different orientations of the hemin and the Arg in both structures. We hypothesize that the developed BP1‐SP1@hemin holds a similar mechanism to the HRP as has been determined before.^[^ ^69^ ^]^ In addition to the SP1 variants, the activity of the peptide sequence of BP1 with hemin was examined. Surprisingly, the peptide sequence of BP1@hemin did not show any enhanced activity in comparison to the cofactor alone (Figure S8, Supporting Information). Also, the BP1@hemin reveals an absorption spectrum similar to hemin (Figure S2, Supporting Information). These results suggest that the SP1 structure is crucial to comprise peroxidase‐like activity. In addition, we examined the activity of a generic protein, bovine serum albumin (BSA), with hemin which showed similar activity to hemin. We postulate that the maximal activity will be obtained for fully coordinated BP1‐SP1. Since each protein structure is built of 12 monomers, a theoretical molar ratio of 1:12 protein to hemin, should allow full binding capacity. To test this hypothesis, the activity of different ratios of BP1‐SP1 to hemin was examined, presenting maximal activity at 1:8. We observed that higher ratios resulted in decreased activity, (Figure S9, Supporting Information). These results may be due to steric interference which affects the proper orientation of the multi‐hemin molecules. Hence, a 1:8 ratio of BP1‐SP1 to hemin was chosen for further analysis.

It should be noted that the natural HRP active site is comprised of three amino acids, His170, Arg38, and His42. The proximal histidine is coordinated with the iron center and acts as a strong electron donor which stabilizes the high oxidation state. The Arg38 and His42 act as proton donors and contribute to the deprotonation of the hydrogen peroxide.^[^ ^68^, ^70^ ^]^ Thus, we concluded that the 1:8 complex provides a microenvironment that better resembles the HRP active site, therefore reaching better activity. Alternatively, the improved activity achieved by the 1:8 complex might be derived from a multi‐coordination of histidines positioned at different SP1 monomers.

Next, the kinetic parameters of the complex were determined. We calculated the BP1‐SP1@hemin catalytic activity rate using a Michalis–Menten‐like kinetic curve, (Figure 2B). The kinetic constants of the BP1‐SP1@hemin and free hemin were determined using Lineweaver–Burk plots as presented in Figure S10 and S11, Supporting Information. The V_max_ and the turnover numbers of the biohybrids are 5‐fold higher than those of the free hemin. The highest rate reached 400 μm min^−1^ as shown in **Table** **1**. It should be noted that while the BP1‐SP1@hemin exhibited improved catalytic activity as compared to hemin, it is still lagging behind the natural peroxidase biocatalytic activity.^[^ ^71^ ^]^ We then examined the catalytic activity of the complex while the H2O2 was kept constant and the ABTS was used as a substrate. Figure S12, Supporting Information, shows the measured catalytic activity. As shown, the affinity of BP1‐SP1@hemin to the ABTS was enhanced showing lower Km values as compared to the H_2_O_2_ substrate. The obtained Km value was 9‐fold lower as compared to the cofactor alone. In addition, while the ABTS was used as a substrate, the *K*_cat_/*K*_m_ value of the hybrid was 6‐fold higher than the cofactor alone, Figure S12, Supporting Information, Table 1. As mentioned, previously developed peroxidase‐like assemblies reached kinetic parameters exceeding those of HRP. For example, Caserta et al.^[^ ^72^ ^]^ have developed a mini‐peroxidase with unnatural amino acids in the peptide sequence. These allowed the binding of the porphyrin to the peptide covalently. Hindson et al.^[^ ^73^ ^]^ have presented a rigidified de novo enzyme with high specificity. The peroxidase mimicry configurations may pave the route toward active site design. The BP1‐SP1@hemin platform that consists of high *K*_m_ values may be beneficial for the oxidation of diverse polymers or molecules, for example, lignin.^[^ ^31^, ^74^, ^75^ ^]^

**Table 1:** Kinetic parameters of hemin, BP1‐SP1@hemin, and HRP

	*K*_m_ [mm]a)	*V*_max_ [μm min^−1^]	*K*_cat_ [min^−1^]	*K*_cat_/*K*_m_ [mm ^−1^ min^−1^]
BP1‐SP1@hemin (H_2_O_2_)	7.2	400	171.7	23.8
Hemin (H_2_O_2_)	1.5	74.6	32.0	20.8
BP1‐SP1@hemin (ABTS)	1.8	212.8	2311.7	1248.5
Hemin (ABTS)	9.8	188.7	2050.0	208.9
HRP^[^ ^97^ ^]^ (H_2_O_2_)	3.7	5.226 (8.71 × 10^−8^ m s^−1^)	2.09 × 10^5^ (3.48 × 10^3^ s^−1^)	5.64 × 10^4^
HRP^[^ ^98^ ^]^ (ABTS)	0.862 (8.62 × 10^−5^ m)		2.562 × 10^4^ (4.27 × 10^2^ s^−1^)	2.97 × 10^4^

a) The kinetic solution contained BP1‐SP1 0.29 μm, hemin 2.3 μm, HEPES buffer 10 mm pH = 8, 10 mm ABTS and 10 mm H_2_O_2_. The reaction was initiated by the addition of H_2_O_2_. The absorbance of ABTS was measured at 650 nm for 1 min. Kinetic parameters were determined by using Lineweaver–Burk plots.

**Figure 3:**
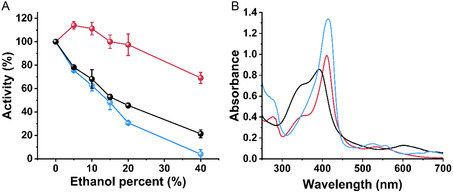
A) Relative activity of BP1‐SP1@hemin complex (red), free hemin (black), and HRP (blue) in different ethanol percentages, H_2_O_2_ 1.28 mm, ABTS 1.28 mm, BP1‐SP1 0.25 μm, hemin 2 μm, HEPES buffer 10 mm pH = 8. The reaction was initiated by the addition of H_2_O_2_. The absorbance of ABTS was measured at 650 nm for 10 min. Error bars represent the standard deviation of independent triplicates. B) The absorbance spectra of BP1‐SP1@hemin complex (red), free hemin (black), and HRP (blue) in 40% ethanol.

Peroxidase is an extremely efficient enzyme that can be utilized in many applications, for example, biosensing.^[^ ^76^ ^]^ As stated earlier, a major obstacle that limits its industrial utilization is low stability. The use of enzymes under harsh conditions at elevated solvent concentrations is highly desirable, as it could lead to shorter processing steps. The SP1 is an extremely stable protein, therefore, we postulate that the BP1‐SP1@hemin will maintain its structure and activity under harsh conditions. For that, the relative activity of the BP1‐ SP1@hemin, free hemin, and HRP were determined under increasing ethanol concentrations. Surprisingly, the BP1‐SP1@hemin complex showed increased activity of 114% and 111% at 5% and 10% ethanol, (**Figure** **3**A). At 15% ethanol, the complex showed 100% activity and a minute decrease to 97% was observed at 20% ethanol. An addition of 40% ethanol, induced a decrease to 69% of the hybrid's original activity. Non‐conjugated hemin presented a sharp activity decline at 40% ethanol reaching 21% of its original activity. Contrary to the BP1‐SP1@hemin, natural horseradish peroxidase was greatly affected by the addition of ethanol, and only 4% of its original catalytic activity was maintained at 40% ethanol. We then examined if the ethanol leads to the disassociation of the hemin from the protein. For that, the spectrum of the samples in 40% ethanol was measured. BP1‐SP1@hemin and hemin did not show any absorbance shift as compared with the complexes in the pure buffer solution. Therefore, we concluded that the hemin does not disassociate from the protein scaffold of the BP1‐SP1@hemin, (Figure 3B, S13, and S14, Supporting Information). In contrast, the HRP spectrum was altered. The soret band at 405 nm was shifted to 414 nm, and new Q‐bands at 527 and 557 nm emerged, (Figure 3B and S15, Supporting Information). This shift was previously observed and attributed to the formation of ferryl HRP intermediates formed by H_2_O_2_.^[^ ^77^, ^78^ ^]^ Since H_2_O_2_ was not present in these samples and the HRP maintained only 4% activity at 40% ethanol, we concluded that the ethanol disrupted the hemin binding site and led to a ferryl‐like inactive compound. It should be noted that while the biohybrid exhibits much better relative stability, the HRP natural activity outperforms the BP1‐SP1@hemin by several orders of magnitudes. We then examined the stability of the biohybrids in acetonitrile. The BP1‐SP1@hemin showed a direct decline in its activity where 85.4% and 80.2% of its activity could be observed in 5% and 10% acetonitrile respectively. 52.9% of its natural activity remained in 40% acetonitrile (Figure S16, Supporting Information). As expected, the HRP stability in acetonitrile was limited, and only 80.5% and 59.6% of its activity was maintained in 5% and 10% of acetonitrile, respectively (Figure S16, Supporting Information). At 40% of acetonitrile, the activity was decreased to 4.3%. Furthermore, the spectrum of the BP1‐SP1@hemin and HRP did not change in elevated acetonitrile concentrations (Figure S17 and S18, Supporting Information). These results suggest that the cofactor is still bonded to HRP and BP1‐SP1 at the elevated acetonitrile concentrations. This important aspect of stability and activity was examined in previous work where an increased activity was gained in 2,2,2‐triflouoroethanol (TFE) solvent.^[^ ^72^, ^73^, ^79^ ^]^

To further examine the complex stability, the absorbance spectra of the BP1‐SP1@hemin complex were followed after heat treatment. As depicted, even at elevated temperatures, the hemin‐protein bond was preserved (Figure S19, Supporting Information). On the contrary, similar measurements performed with the natural HRP have exhibited small shifts at the 500 nm and 650 nm peaks (Figure S20, Supporting Information). These results can be attributed to structural changes occurring at high temperatures in the native HRP.

**Figure 4:**
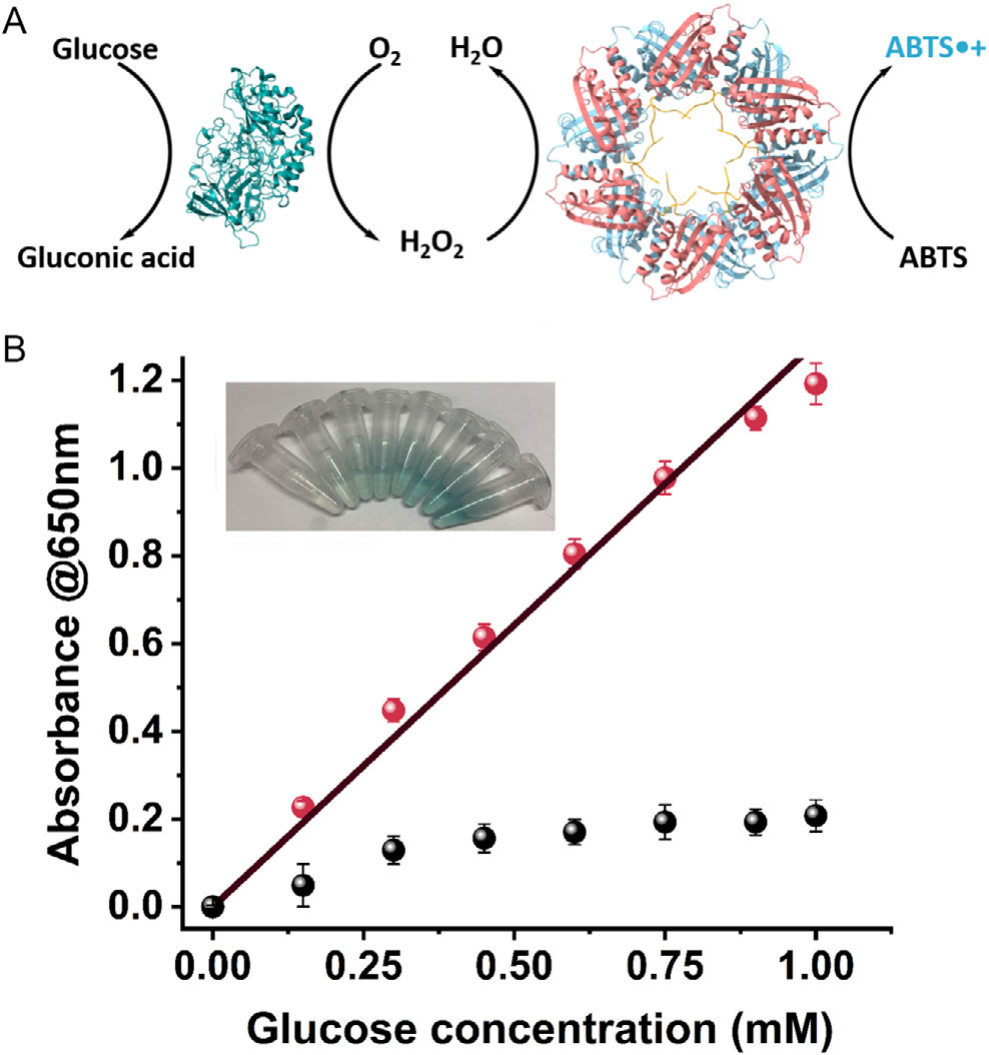
A) Glucose biosensor scheme based on BP1‐SP1@hemin (PDB code 1TR0) complex and GO*x* (PDB code 1CF3). B) The performance of the glucose biosensor, showing the BP1‐SP1@hemin complex (red dots), and free hemin (black dots). 5 mm ABTS, 0.2 μm GO*x*, 5 μm hemin, 0.625 μm BP1‐SP1, HEPES buffer 10 mm pH = 8. The reaction was initiated by glucose addition at 37 °C and the absorbance was measured at 650 nm for 10 min. Termination was achieved by the addition of 100% DMSO. Inset, a photograph of the glucose biosensor samples in increasing glucose concentrations. Error bars represent the standard deviation of independent triplicates.

HRP and “HRP‐like” complexes are commonly utilized with oxidases to enable the optical detection of analytes or for the healing of infected wounds.^[^ ^80^, ^81^, ^82^, ^83^, ^84^ ^]^ Therefore, we examined if the biohybrid could be used for biosensing. For that, the biohybrid was coupled with glucose oxidase (GO*x*) to develop a glucose optical biosensing test, (**Figure** **4**A). The reaction was initiated by the addition of glucose, which in turn was oxidized by the GO*x* to generate gluconic acid and hydrogen peroxide. In turn, the hydrogen peroxide and the chromogenic agent (ABTS) were catalyzed by the artificial peroxidase to yield an optical signal that can be followed spectroscopically.^[^ ^85^ ^]^ A linear correlation between the UV/Vis absorbance and the different glucose concentrations between 0 and 1 mm could be obtained, with limit of detection (LOD) of 0.055 mm (Figure 4B). Replacing the BP1‐SP1@hemin with hemin resulted in poor sensing range and activity, (Figure 4B). The obtained LOD and sensing range is similar to other peroxidase mimicking assemblies.^[^ ^86^, ^87^, ^88^, ^89^ ^]^ These results demonstrate the potential of the developed platform for future applications. The use of peroxidase‐like enzymes for sensing under nonaqueous solutions may open the path for continuous monitoring apparatus that operate in the industrial conditions, which are currently too harsh for natural enzymes.

**Figure 5:**
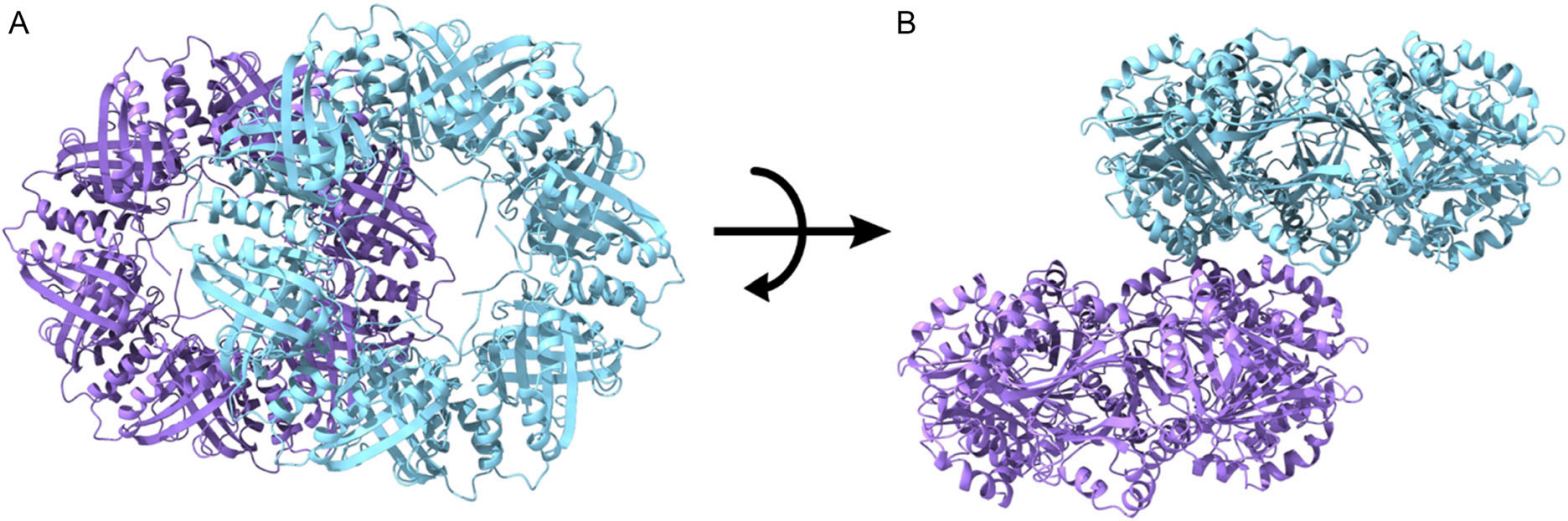
BP1‐SP1@hemin crystal structure at a resolution of 2.4 Å. A top view A) and a side view B) of the two rings in the asymmetric unit. (B).

While the BP1‐SP1@hemin complex was imaged by TEM and examined by DLS and spectral absorbance, our results did not provide any structural data, and the hemin orientation and binding site(s) were not determined. This valuable information can provide the roadmap for improved rational biohybrid design. Toward this goal, the BP1‐SP1@hemin complex, BP1‐SP1, and WT‐SP1 were crystallized and their structures were determined as described in the Materials and Methods section. The X‐ray crystallographic structure of WT‐SP1 was determined at 3.1 Å resolution. The crystal belongs to the tetragonal space group I422 with a trimer in the asymmetric unit, as reported previously.^[^ ^53^ ^]^ The dodecamer ring structure can be visualized by crystallographic symmetry operations, (Figure S21A, Supporting Information). Interestingly, differences in the crystal lattice packing were observed compared to the previous WT‐reported structure, perhaps due to the minor differences in the crystallization conditions. The rings are organized in the crystal in a fashion that brings the circumference of one ring close to the aperture of the second ring. The rings are located perpendicular to each other, leading to a tightly condensed packing with limited free space inside the ring central cavity, (Figure S21B, Supporting Information). This structure differs from the WT crystal packing that was described before and from the BP1‐SP1@hemin. Two crystallization trials were performed for the BP1‐SP1@hemin complex. The first was performed with the previously established WT‐SP1 crystallization conditions^[^ ^53^ ^]^ while the second was crystallized as described in the materials and methods section. The hexagonal crystals, diffracted to a resolution of 2.4 Å, belong to the monoclinic space group C2 with a hexamer at the asymmetric unit. A ring‐like structure can be visualized by crystallographic symmetry operations. The rings are oriented in one continuous plane, unlike the perpendicular WT‐SP1 structure (data not shown). The larger crystals obtained by the second trial, (Figure S22, Supporting Information), diffracted to a resolution of 2.4 Å and belong to the monoclinic space group P21 with two dodecamers in the asymmetric unit. Each dodecamer forms a ring‐like structure, (**Figure** **5**), with a different packing than that found in the WT structure, (Figure S21, Supporting Information). Clear electron density was observed for the ring‐like shape structure, (Figure S23A, Supporting Information). While the crystal structure of the BP1‐SP1@hemin complex is identical to the WT‐SP1, (root mean square deviation = 0.278 Å, Figure S24, Supporting Information), suggesting that SP1 self‐assembly was not affected. The two rings in the asymmetric unit are shifted relative to each other, forming a structure of staggered layers.

This different crystal packing compared to the WT, (Figure S21, Supporting Information) may be explained by the N‐terminal extensions of the BP1‐SP1. The structure leaves enough free space for the N‐terminal extension to stick out the ring since the extensions of 15 residues are too long to be located inside the ring central cavity (unlike the WT structure which lacks those extensions). We attribute these results to the high flexibility of the N‐terminal extension. Consequentially no electron density was found inside the central cavity that could be modeled for those residues or the hemin molecule, (Figure S23B, Supporting Information). Therefore, it is highly likely that the hemin can bind at several positions of the BP1‐SP1 N‐terminal extensions. Therefore, heterogeneity in the hemin binding was achieved, leading to poor electron density maps. This is in agreement with the previously determined WT SP1 structure, where the first two amino acids within the inner pore could not be modeled.^[^ ^53^ ^]^ Taking into consideration the change in spectrum upon the complexation of hemin with the BP1‐SP1, the inability to identify hemin could be a consequence of the crystallization process that affects coordination. Interestingly, an attempt to stabilize the BP1‐SP1@hemin structure by pre‐cooling the crystals to 4 °C before the freezing process did not lead to improved electron density due to the hemin interaction, alluding to variability in positions of the N‐terminal tails and hemin binding positions. Ni et al.^[^ ^90^ ^]^ have introduced a new strategy to determine the structure of flexible peptides by encapsulating them into an ordered protein lattice. In our case, the active residues coordinated with the hemin site, are not surrounded by the SP1's ordered lattice. Hence, the inability to determine the structure of the N‐terminus is conceivable.

It should be noted that the BP1‐SP1 alone exhibited the same structure and asymmetric unit as the BP1‐SP1@hemin complex, strengthening the conclusion that the different packaging of the BP1‐SP1 is due to the added N‐terminus, (Figure S25 and S26, Supporting Information).

## 3 Conclusion

In this work, we present a new stable supramolecular scaffold that consists of hemin cofactor as a model for the development of artificial enzymes. Toward this goal, we examined the suitability of the SP1 proteins’ unique scaffold to act as an outer‐sphere building block for an enzyme‐like active site. This approach opens new routes to design artificial enzymes for natural and non‐natural reactions at varied conditions. The extremely high stability of the SP1 adds many possibilities for its use in many future applications. As a proof of concept, we have coupled the developed biohybrid with the GO*x* enzyme to construct an optical glucose biosensor. The BP1‐SP1@hemin complex presents lower catalytic activity in comparison to the natural peroxidase, however, can maintain its activity in the presence of elevated ethanol concentrations. Computational methodologies are commonly used for enzyme design and have high importance for future catalysts’ construction. Nevertheless, we believe that using a stable outer‐sphere structure could enable an easier practice to bioengineer a desired active site environment comprised of different co‐factors or binding peptides. This may enable higher rates or drive desired unnatural reactions.

## 4 Experimental Section

4.1

4.1.1

##### Construction of Modified BP1‐SP1, BP2‐SP1, BP3‐SP1, and the WT‐SP1

The variant BP4‐SP1 constructed in pJexpress 411 T7 Kan (ATUM) plasmid including the restriction sites of NdeI and BamHI in the N‐terminus and C‐terminus respectively. The plasmid was kindly given by Oded Shoseyov's group, at the Hebrew University of Jerusalem and purified from *E. coli* BL21 (DE3). In order to obtain the BP1‐SP1, BP2‐SP1, BP3‐SP1, and the WT sequences, the BP4‐SP1 was altered using molecular biology tools. For the BP3‐SP1 and the WT‐SP1 sequences, overhang PCR with forward primers including the bases encoding for BP3, NdeI, and BamHI restriction sites were used. The BP1‐SP1 sequence was amplified by PCR from an ordered pUC57 plasmid (Bio Basic). The BP2‐SP1 gene was ordered as a g‐block with the same NdeI and BamHI restriction sites. All used primer sequences are presented in the supporting information. After amplification, each of the BP3‐SP1, WT‐SP1, and BP1‐SP1 sequences was digested and purified from a gel using NucleoSpin PCR clean‐up kit (MACHEREY‐NAGEL). The BP2‐SP1 was digested directly and added to the predigested pJexpress 411 T7 Kan plasmid and further ligated. Heat shock was performed for plasmid transformation to *E. coli* DH5*α* competent cells. The bacteria were grown on selective agar media containing kanamycin. Colonies were picked and sent for colony‐PCR assay using specific primers for BP1‐SP1, BP2‐SP1, BP3‐SP1, and WT‐SP1 to ensure the insertion of the desired sequence. Positive colonies were grown on LB plates and further analyzed by Sanger Sequencing. The verified plasmids were transformed to BL21 (DE3) competent cells.

##### SP1 Variants Extraction

A starter of *E. coli* BL21 with the desired plasmid was prepared from glycerol stock in a 5 mL LB medium. The bacteria were incubated and shaken at 37 °C, 180 rpm overnight. The next day, 760μL of the starter was transferred to a 0.5 L LB medium, incubated at 37 °C, 180 rpm. When the medium reached an OD_600nm_ ≈ 0.6, 0.1 mm IPTG was added to induce protein overexpression at 23 °C, 220 rpm (overnight). The cells were further centrifuged at 4500 rpm for 40 min. Then, the bacteria were suspended in 12 mL 10 mm PB pH = 7.4. Lysis was performed using a Qsonica Q500‐ 500 W sonicator, 40% amplitude, 5 on, 5 s off cycles for 10 min. The gained cell lysate was further centrifuged (x2) for 15 min at 11 000 rpm at 4 °C. The supernatant was collected and further purified using anion exchange chromatography (Hiprep DEAE FF 16/10, GE Healthcare), using 10 mm PB pH = 7.4 buffer, with NaCl gradient between 25 and 500 mm. The fraction containing the SP1 was then purified using a 25 mL size exclusion column (Superdex 200 increase 10/300 GL, GE Healthcare) with 10 mm PB, and 25 mm NaCl buffer. To eliminate salt excess, the fraction was filtered against a dialysis membrane (MWCO 12‐14 kDa, REPLIGEN) with 10 mm HEPES. Protein concentration was determined using UV‐Vis spectroscopy at 280 nm. SDS‐PAGE gels (SurePAGE, Bis‐Tris 4%‐20%, GenScript) were used to determine the protein purity and size. For the BP4‐SP1 extraction, the same lysis procedure was performed, however, the cell lysate was loaded on immobilized metal affinity chromatography (1 mL, High‐affinity Ni‐NTA resin, GenScript). The column was washed with 0.1 m PB, 500 mm NaCl, 50 mm imidazole, and pH = 7.4 as an equilibrium buffer. To elute the protein, the column was washed with 0.1 m PB, 500 mm NaCl, and 500 mm imidazole pH = 7.4 as an elution buffer.

##### Modified SP1@hemin Complexes Preparation

500 μm hemin stock solution was prepared by dissolving hemin in 100% DMSO. From this stock, 50 μm hemin stock solution was prepared by diluting the previous solution in 10 mm HEPES pH = 8. To prepare the complex, 20 μm of hemin was mixed with the desired concentration of SP1 and further incubated for 1 h at room temperature. To eliminate excess unbound hemin, the samples were purified using a desalting microspin column (PD SpinTrap G‐25, Cytiva) using 10 mm HEPES as an equilibrium buffer. The biohybrids were stored at 4C and showed stability for months.

##### TEM Analysis of BP1‐SP1@hemin

5 μL of the 1:8 BP1@SP1 solution (BP1‐SP1 1.25 μm, hemin 10 μm) was dropped on a carbon‐coated copper grid. Blotting paper was used to eliminate excess solution. To stain the protein, 5 μL of 1% uranyl acetate was added. After removing the extra solution, the grid was allowed to dry at room temperature. The grid was stored under a vacuum until the microscope session.

##### Activity Assay

The assay was performed in activity solution buffer, HEPES 10 mm pH = 8. A 1.28 mm H_2_O_2_ (dissolved in water), 1.28 mm ABTS (dissolved in 10 mm HEPES pH = 8). The modified SP1@hemin complex was then added (volume was corrected while needed), resulting in a final concentration of 2 μm hemin. The reaction was initiated by an addition of H_2_O_2_. The reaction was followed by the ABTS probe using UV‐Vis spectroscopy at 650 nm. The activity of the SP1@hemin complex in organic solvents was monitored in the same fashion, however, due to different kinetics parameters, the HRP examination was performed with 1.5 nm. Similarly to the aqueous measurements, the reaction was initiated by the addition of H_2_O_2_. The relative activity of the samples was calculated using the following equation.

(1) $$V=\frac{\left({\text{Abs}}_{t=\;1\text{min}}-{\text{Abs}}_{t=\;0\text{min}}\right)}{t\cdot \varepsilon_{650\text{ nm}}\cdot l}$$

*V* ‐ Velocity

Abs_*t* = 1 min_ ‐ Absorbance after one minute

Abs_*t* = 0 min_ ‐ Absorbance at time zero

*ε*_650nm_ ‐ Extension coefficient of ABTS at 650 nm

*l* ‐ Path length

*t* – Time

##### Glucose Biosensor

The solution was prepared in 10 mm HEPES buffer pH = 8. The solution contained 5 μm hemin, 0.625 μm SP1, 5 mm ABTS, 0.2 μm GO*x*, and different glucose concentrations. The reaction was initiated by the addition of glucose and ran for 10 min at 37 °C. In order to terminate the reaction, 100% DMSO *δ* (100 μL) was added to each sample. The linear range of glucose sensing can be described by the trend: *y* = 1.19*x* + 0.05. The detection limit may be expressed using the following trend.^[^ ^91^ ^]^

(2) $$\text{Limit of detection}=\frac{3.3\cdot \delta }{S}$$

*δ*‐Standard deviation of the response

*S*‐Calibration curve slope

##### Crystallization and Data Collection of WT‐SP1

Crystallization trials of the WT sample were initiated with the previously determined WT‐SP1 crystallization conditions.^[^ ^53^ ^]^ Since crystals were not obtained, screening was performed including other types of PEG. Rod crystals of WT‐SP1 were obtained by hanging drop vapor diffusion method within 7 days at 20 °C. 2 μL of protein solution (7 mg mL^−1^ in 50 mm Tris pH = 7.5, 100 mm NaCl, 20 mm CaCl_2_) were mixed with 2 μl of reservoir solution containing 0.1 m HEPES pH = 7.5, 0.2 N NaCl and 20% PEG 3350. The obtained crystals were mounted on a cryo‐loop (CrystalCap SPINE HT, Hampton Research) and flash‐frozen in liquid nitrogen. X‐ray diffraction data was collected at the European Synchrotron Radiation Facility (ESRF) on beamline ID30‐B. Diffraction data was integrated with XDS^[^ ^92^ ^]^ and processed with CCP4.^[^ ^93^ ^]^

The X‐ray crystallographic structure of WT‐SP1 was determined by molecular replacement (MR) using the WT‐SP1 (PDB:1SI9) as the homology model. The resulting solution was refined and rebuilt using phenix.refine^[^ ^94^ ^]^ and crystallographic object‐oriented toolkit (COOT).^[^ ^95^ ^]^ Statistics for diffraction data collection, structure determination, and refinement are summarized in Table S1, Supporting Information.

##### A Crystallization and Data Collection of BP1‐SP1@hemin

Crystallization trials of the BP1‐SP1‐hemin sample were performed with the previously established WT‐SP1 crystallization conditions.^[^ ^53^ ^]^ For this variant protein, the crystallization liquor‐induced precipitation and screening for different crystallization conditions were done by robotic methods at the Technion Center for Structural Biology (TCSB) using commercially available screens (Hampton Research). Hexagonal crystals of BP1‐SP1 were obtained by hanging drop vapor diffusion method at 20 °C for 2 days. 0.15 μL of protein solution (7 mg mL^−1^ in 0.1 m HEPES pH = 8.0, 2.2% DMSO) was mixed with 0.15 μL of reservoir solution containing 2% v/v Tacsimate pH = 7, 0.5% v/v 2‐Propanol, 0.1 m Imidazole pH = 7.0, 8% w/v PEG 3350. The obtained crystals were mounted on a cryo‐loop (CrystalCap SPINE HT, Hampton Research) and flash‐frozen in liquid nitrogen. X‐ray diffraction data was collected at the European Synchrotron Radiation Facility (ESRF) on beamline ID30A‐3. Diffraction data was integrated with XDS^[^ ^92^ ^]^ and processed with CCP4.^[^ ^93^ ^]^ The X‐ray crystallographic structure of BP1‐SP1 was determined by molecular replacement (MR) using the WT‐SP1 (PDB:1SI9) as the homology model. The resulting solution was refined and rebuilt using phenix.refine^[^ ^94^ ^]^ and COOT.^[^ ^95^ ^]^

Larger orange crystals, (Figure S22, Supporting Information) were obtained by hanging drop vapor diffusion method at 20 °C in 7 days. 2 μL of protein (7 mg mL^−1^ in 0.1 m HEPES pH = 8.0, 2.2% DMSO) was mixed with 2 μL of reservoir solution containing 7.5% v/v Tacsimate pH 7.0, 5% v/v 2‐Propanol, 0.1 m Imidazole pH = 7.0, 13% w/v PEG 3350 (being an optimized condition following PEGrx screen, Hampton Research). The crystals were mounted on a cryo‐loop (CrystalCap SPINE HT, Hampton Research) and flash‐frozen in liquid nitrogen. X‐ray diffraction data was collected at the European Synchrotron Radiation Facility (ESRF) on beamline ID23‐2. Diffraction data was integrated with XDS^[^ ^92^ ^]^ and processed with CCP4.^[^ ^93^ ^]^

The X‐ray crystallographic structure of BP1‐SP1 was determined by molecular replacement (MR) using the WT SP1 ring (PDB: 1TR0) as the homology model. The resulting solution was refined and rebuilt using phenix.refine^[^ ^94^ ^]^ and COOT.^[^ ^95^ ^]^ Statistics for diffraction data collection, structure determination, and refinement are summarized in Table S1, Supporting Information.

##### Crystallization and Data Collection of BP1‐SP1

Screening for crystallization conditions was done by robotic methods at the Technion Center for Structural Biology (TCSB) using commercially available screens (Hampton Research). Crystals of BP1‐SP1 that diffracted to a resolution of 2.4 Å were obtained by hanging drop vapor diffusion method at 20 °C in 7 days. Crystals were obtained by mixing 2 μL of protein solution (7 mg mL^−1^ in 0.1 m HEPES pH = 8.0) with 2 μL of reservoir solution containing 0.2 m Potassium sodium tartrate tetrahydrate, 20% PEG 3350 (Index screen, Hampton Research). The obtained crystals were mounted on a cryo‐loop (CrystalCap SPINE HT, Hampton Research) and flash‐frozen in liquid nitrogen. X‐ray diffraction data was collected at the European synchrotron radiation facility (ESRF) on beamline ID23‐2. Diffraction data was integrated with XDS^[^ ^92^ ^]^ and processed with CCP4.^[^ ^93^ ^]^

The X‐ray crystallographic structure of BP1‐SP1 was determined by molecular replacement (MR) using the WT‐SP1 ring (PDB:1TR0) as the homology model. The resulting solution was refined and rebuilt using phenix.refine^[^ ^94^ ^]^ and COOT.^[^ ^95^ ^]^ Statistics for diffraction data collection, structure determination, and refinement are summarized in Table S1, Supporting Information. The final structures were deposited in the PDB under the accession codes 8OZ4, 8OZO, and 8OZS.

## Conflict of Interest

The authors declare no conflict of interest.

## Supporting information

Supplementary Material

## Data Availability

The data that support the findings of this study are available from the corresponding author upon reasonable request.
