# Supplementary material for: A Bioengineered Stable Protein 1‐Hemin Complex with Enhanced Peroxidase‐Like Catalytic Properties
Source: Small Sci. 2024 Mar 19;4(6):2400025. doi: 10.1002/smsc.202400025 (PMC11935090; doi:10.1002/smsc.202400025)
Supplement: Supplementary file 1 — Supplementary Material [file SMSC-4-2400025-s001.pdf]

Supporting Information

A bioengineered SP1-hemin biohybrid mimics the activity of peroxidase with improved characteristics

Yara Zeibaq,[a] *Oren Bachar,*[a] *Jenia Sklyar,*[b] *Noam Adir,*[b] *and Omer Yehezkeli* [*a,c,d]

Table of Contents

Contents

Table S1. Data collection and refinement statistics. 3

- WT-SP1 3
- BP1-SP1 3
- BP1-SP1@hemin 3

SP1 variant protein sequences 4

SP1 variant DNA sequences 4

Supplementary figures 6

Table S1. Data collection and refinement statistics.

	WT-SP1 (PDB: 8OZ4)	BP1-SP1 (PDB: 8OZO)	BP1-SP1@hemin (PDB: 8OZS)
**Wavelength (Å)**	0.976253	0.873128	0.873128
**Resolution range (Å)**	80.76- 3.10 (3.41-3.1)	48.16- 2.40 (2.43-2.40)	48.60- 2.40 (2.44-2.40)
**Space group**	I 4 2 2	P 1 21 1	P 1 21 1
**Unit cell**	89.573 89.573 186.746 90 90 90	96.839 94.458 167.955 90 90.068 90	97.059 94.372 168.069 90 90.224 90
**Total reflections**	135399 (15006)	658515 (32924)	656871 (33472)
**Unique reflections**	7263 (1289)	118409 (5849)	118649 (5863)
**Multiplicity**	18.7 (11.6)	5.6 (5.6)	5.5 (5.7)
**Completeness (%)**	99.8 (99.9)	99.8 (99.8)	99.8 (99.9)
**Mean I/sigma(I)**	14.6 (4.0)	7.5 (2.7)	6.3 (2.8)
**Wilson B-factor (Å^2)**	67.55	20.60	15.63
**R-merge**	0.138 (0.483)	0.096 (0.341)	0.115 (0.344)
**R-meas**	0.145 (0.527)	0.117 (0.415)	0.140 (0.418)
**R-pim**	0.033 (0.150)	0.066 (0.234)	0.080 (0.235)
**CC1/2**	0.995 (0.975)	0.998 (0.968)	0.996 (0.955)
**Reflections used in refinement**	7114 (3112)	117956 (3729)	118235 (3758)
**Reflections used for R-free**	602 (125)	5904 (207)	5992 (213)
**R-work**	0.2200 (0.3298)	0.1819 (0.2291)	0.1847 (0.2290)
**R-free**	0.2875 (0.4507)	0.2374 (0.2949)	0.2376 (0.2874)
**Number of non-hydrogen atoms**	2593	22708	22630
**Macromolecules**	2593	20712	20702
**Ligands**	0	0	0
**Solvent**	0	1996	1928
**Protein residues**	312	2496	2496
**RMS (bonds, Å)**	0.010	0.007	0.007
**RMS (angles, °)**	1.18	0.86	0.85
**Ramachandran favored (%)**	97.79	99.04	99.24
**Ramachandran allowed (%)**	3.21	0.96	0.76
**Ramachandran outliers (%)**	0.00	0.00	0.00
**Rotamer outliers (%)**	0.35	0.71	0.27
**Clashscore**	7.44	4.37	4.39
**Average B-factor (Å^2)**	87.57	23.62	23.70

Statistics for the highest-resolution shell are shown in parentheses.

SP1 variant protein sequences

WT-SP1

MATRTPKLVKHTLLTRFKDEITREQIDNYINDYTNLLDLIPSMKSFNWGTDLGMESAEL
NRGYTHAFESTFESKSGLQEYLDSAALAAFAEGFLPTLSQRLVIDYFLY

BP1-SP1

MMHGKTQATSGTIQSATRTPKLVKHTLLTRFKDEITREQIDNYINDYTNLLDLIPSMKSF
NWGTDLGMESAELNRGYTHAFESTFESKSGLQEYLDSAALAAFAEGFLPTLSQRLVIDY
FLY

BP2-SP1

MGDVHHHGRHGAEHADIATRTPKLVKHTLLTRFKDEITREQIDNYINDYTNLLDLIPSM
KSFNWGTDLGMESAELNRGYTHAFESTFESKSGLQEYLDSAALAAFAEGFLPTLSQRLV
IDYFLY

BP3-SP1

MSVTQNKYATRTPKLVKHTLLTRFKDEITREQIDNYINDYTNLLDLIPSMKSFNWGTDL
GMESAELNRGYTHAFESTFESKSGLQEYLDSAALAAFAEGFLPTLSQRLVIDYFLY

BP4-SP1

MHHHHHHATRTPKLVKHTLLTRFKDEITREQIDNYINDYTNLLDLIPSMKSFNWGTDLG
MESAELNRGYTHAFESTFESKSGLQEYLDSAALAAFAEGFLPTLSQRLVIDYFLY

SP1 variant DNA sequences

WT-SP1

ATG GCC ACC CGC ACT CCG AAA CTG GTA AAA CAT ACG CTG CTT ACT CGC TTT
AAG GAT GAA ATT ACC CGC GAG CAG ATT GAT AAC TAT ATC AAT GAT TAT ACA
AAT CTT TTA GAC TTG ATT CCC AGC ATG AAG TCA TTT AAC TGG GGT ACA GAT
CTT GGT ATG GAG TCA GCC GAG CTG AAC CGT GGC TAC ACC CAC GCC TTT GAA
TCG ACA TTC GAG TCG AAA TCA GGT TTA CAA GAA TAC CTG GAC TCT GCC GCC
TTG GCA GCA TTC GCC GAG GGA TTC TTA CCT ACT CTG AGT CAA CGC TTA GTC
ATT GAC TAC TTC TTG TAT

BP1-SP1

ATG ATG CAT GGC AAA ACC CAG GCG ACT TCT GGA ACA ATC CAA AGT GCC ACC
CGC ACT CCG AAA CTG GTA AAA CAT ACG CTG CTT ACT CGC TTT AAG GAT GAA
ATT ACC CGC GAG CAG ATT GAT AAC TAT ATC AAT GAT TAT ACA AAT CTT TTA
GAC TTG ATT CCC AGC ATG AAG TCA TTT AAC TGG GGT ACA GAT CTT GGT ATG

GAG TCA GCC GAG CTG AAC CGT GGC TAC ACC CAC GCC TTT GAA TCG ACA TTC GAG TCG AAA TCA GGT TTA CAA GAA TAC CTG GAC TCT GCC GCC TTG GCA GCA TTC GCC GAG GGA TTC TTA CCT ACT CTG AGT CAA CGC TTA GTC ATT GAC TAC TTC TTG TAT

BP2-SP1

ATG GGC GAC GTA CAC CAC CAC GGT CGT CAT GGT GCA GAA CAC GCA GAT ATT GCC ACC CGC ACT CCG AAA CTG GTA AAA CAT ACG CTG CTT ACT CGC TTT AAG GAT GAA ATT ACC CGC GAG CAG ATT GAT AAC TAT ATC AAT GAT TAT ACA AAT CTT TTA GAC TTG ATT CCC AGC ATG AAG TCA TTT AAC TGG GGT ACA GAT CTT GGT ATG GAG TCA GCC GAG CTG AAC CGT GGC TAC ACC CAC GCC TTT GAA TCG ACA TTC GAG TCG AAA TCA GGT TTA CAA GAA TAC CTG GAC TCT GCC GCC TTG GCA GCA TTC GCC GAG GGA TTC TTA CCT ACT CTG AGT CAA CGC TTA GTC ATT GAC TAC TTC TTG TAT

BP3-SP1

ATG TCC GTC ACC CAG AAC AAG TAC GCC ACC CGC ACT CCG AAA CTG GTA AAA CAT ACG CTG CTT ACT CGC TTT AAG GAT GAA ATT ACC CGC GAG CAG ATT GAT AAC TAT ATC AAT GAT TAT ACA AAT CTT TTA GAC TTG ATT CCC AGC ATG AAG TCA TTT AAC TGG GGT ACA GAT CTT GGT ATG GAG TCA GCC GAG CTG AAC CGT GGC TAC ACC CAC GCC TTT GAA TCG ACA TTC GAG TCG AAA TCA GGT TTA CAA GAA TAC CTG GAC TCT GCC GCC TTG GCA GCA TTC GCC GAG GGA TTC TTA CCT ACT CTG AGT CAA CGC TTA GTC ATT GAC TAC TTC TTG TAT

BP4-SP1

ATG CAC CAT CAC CAC CAC CAC GCC ACC CGC ACT CCG AAA CTG GTA AAA CAT ACG CTG CTT ACT CGC TTT AAG GAT GAA ATT ACC CGC GAG CAG ATT GAT AAC TAT ATC AAT GAT TAT ACA AAT CTT TTA GAC TTG ATT CCC AGC ATG AAG TCA TTT AAC TGG GGT ACA GAT CTT GGT ATG GAG TCA GCC GAG CTG AAC CGT GGC TAC ACC CAC GCC TTT GAA TCG ACA TTC GAG TCG AAA TCA GGT TTA CAA GAA TAC CTG GAC TCT GCC GCC TTG GCA GCA TTC GCC GAG GGA TTC TTA CCT ACT CTG AGT CAA CGC TTA GTC ATT GAC TAC TTC TTG TAT

Table S2: Primers used for the construction of the different plasmids

Primer		Sequence
WT-SP1	Forward	GATA**CATATG**GCAACCAGAACTCCAAAACTTGTGAAGCACA
	Reverse	GCTT**GGATCC**TTAGTAGAGAAAATAGTCTATCACAAGACGC
BP1-SP1	Forward	GATA**CATATG**ATGCATGGCAAAACC
	Reverse	CATCTAGATATC**GGATCC** TTAGTAGAG
BP3-SP1	Forward	GATA**CATATG**AGCGTGACCCAGAACAAATATGCAACCAGA<br>ACTCAAAACTTGTGAAGCACACATTG
	Reverse	GCTT**GGATCC**TTAGTAGAGAAAATAGTCTATCACAAGA<br>CGCTGTGACAAAGTAGGCAAAAACCC

- Bases in red are the restriction sites of NdeI and BamHI

Supplementary figures

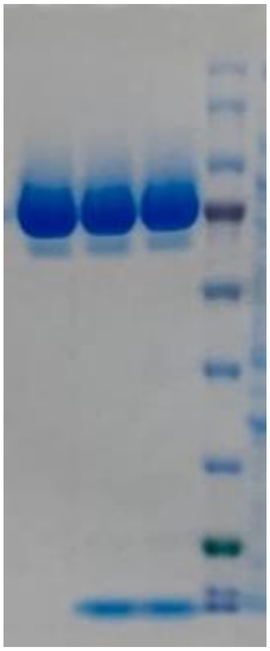

Figure S1. SDS-PAGE analysis of the purified BP1-SP1. The samples were mixed SDS–PAGE sample buffer by using and heated at 95°C for 10min.

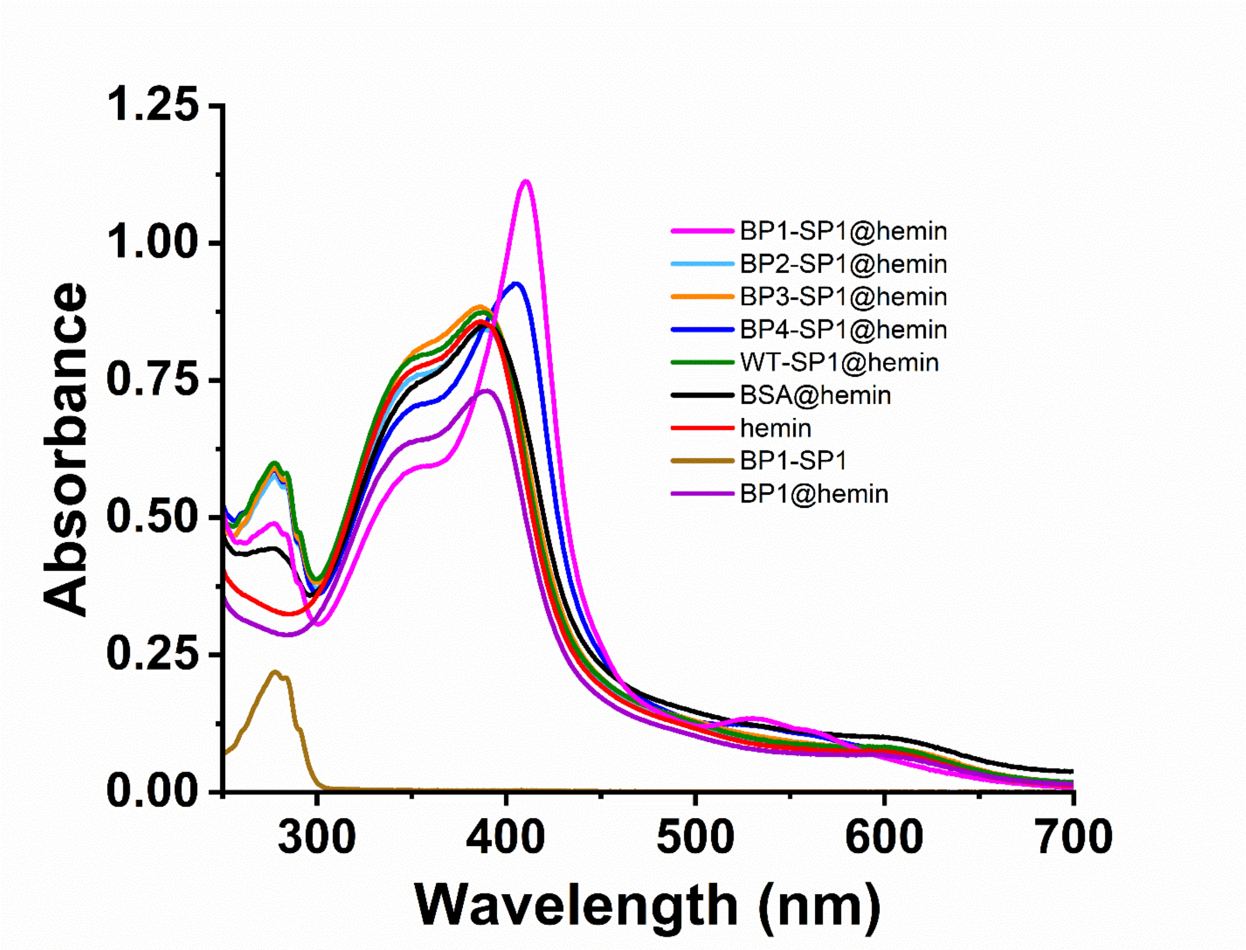

Figure S2. Absorption spectrum of the different SP1 variants with hemin. Protein concentration of all the biohybrids is 2.5µM. Hemin concentration in all the biohybrids is 20µM. The biohybrids and hemin solution consists of 95% HEPES and 5% DMSO

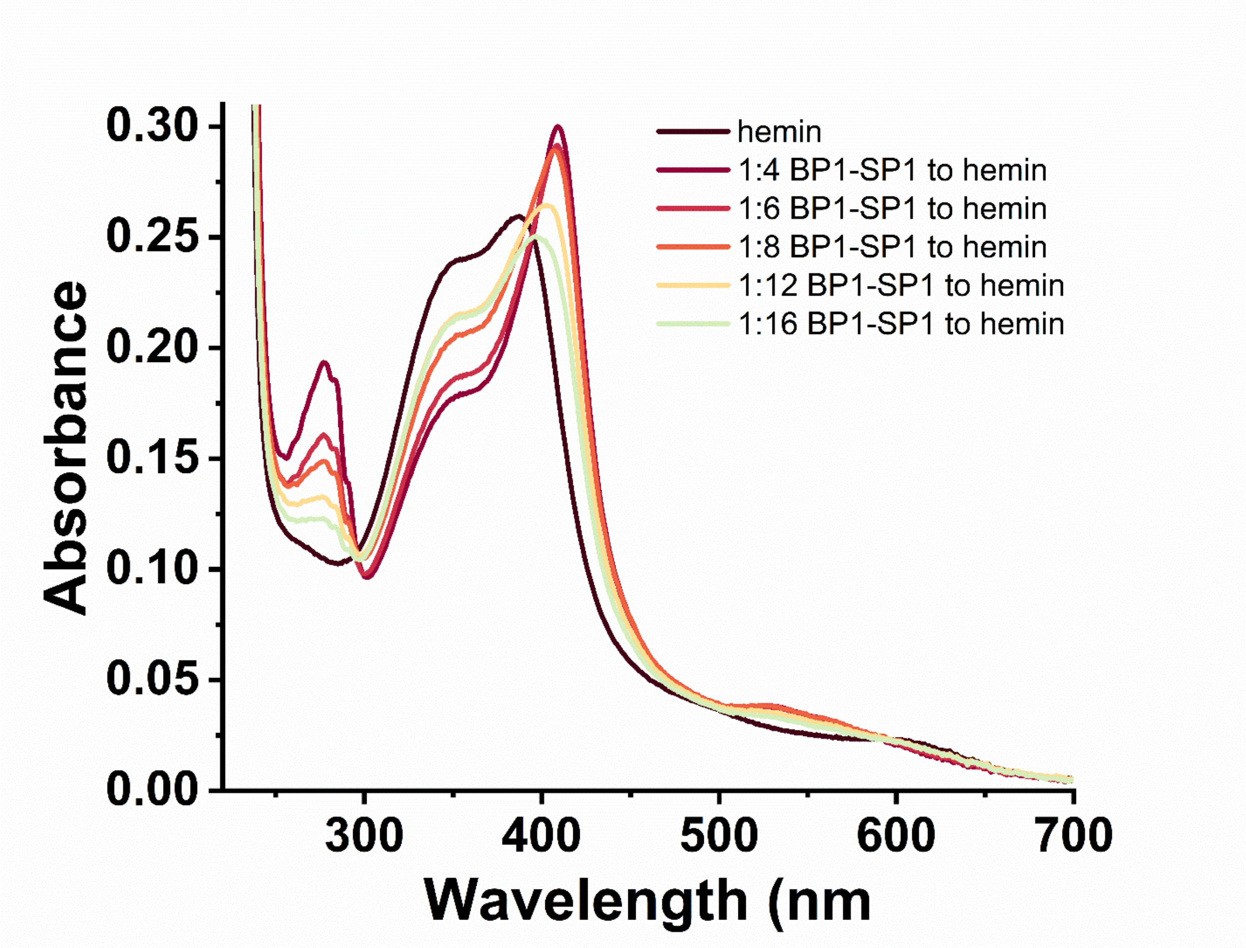

Figure S3. Absorption spectra of the BP1-SP1@hemin complex at different protein to hemin ratios. All of the samples contained hemin 20μM. The protein concentration was varied from 1.25μM to 5μM. The biohybrids and hemin solution consists of 95% HEPES and 5% DMSO

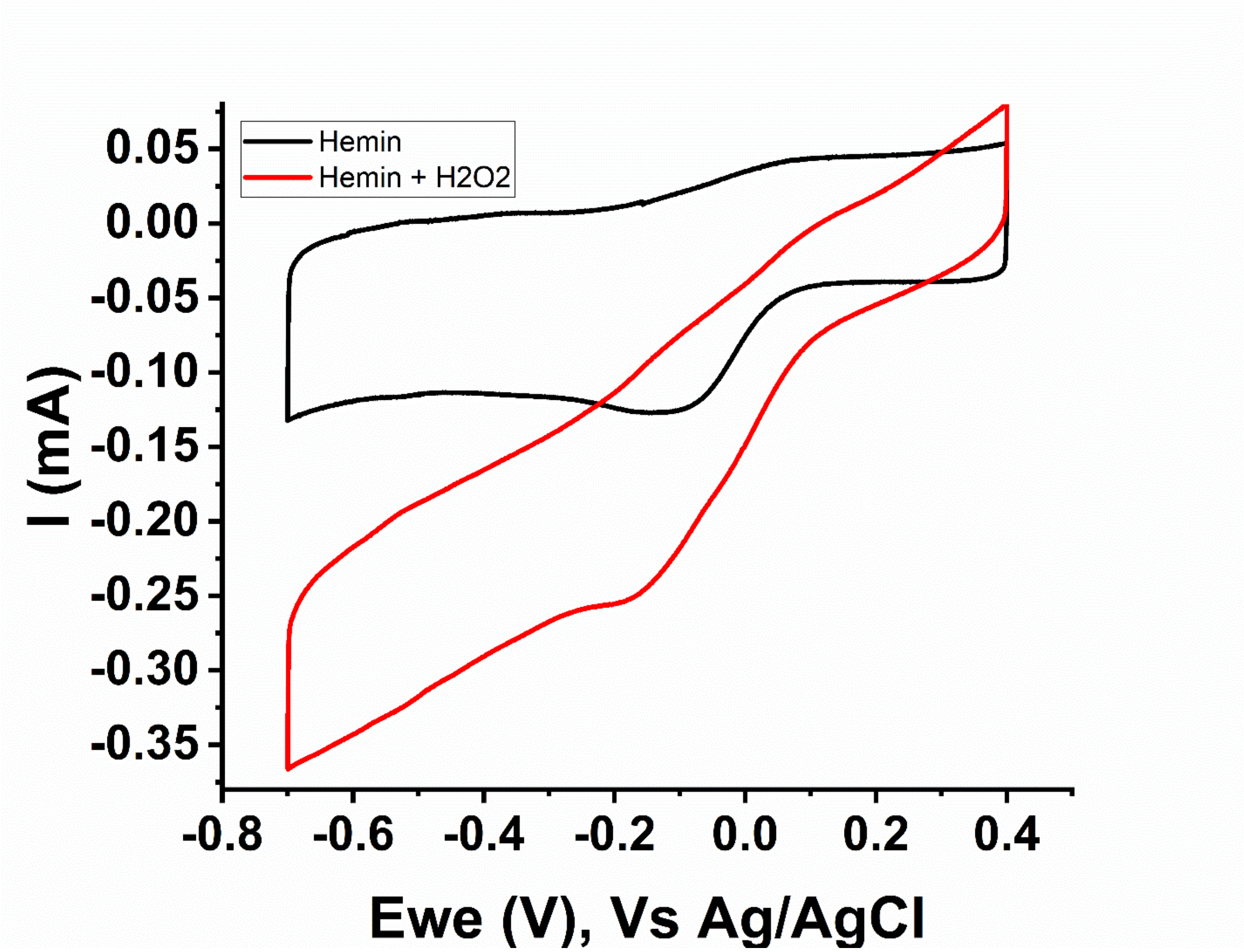

Figure S4. Cyclic voltammogram of hemin in 95% 10mM HEPES and 5% DMSO at glassy carbon electrode with and without H_2O_2. The measurement was performed in PB 0.1 M, pH = 7.5 at 5 mV/s in anaerobic conditions.

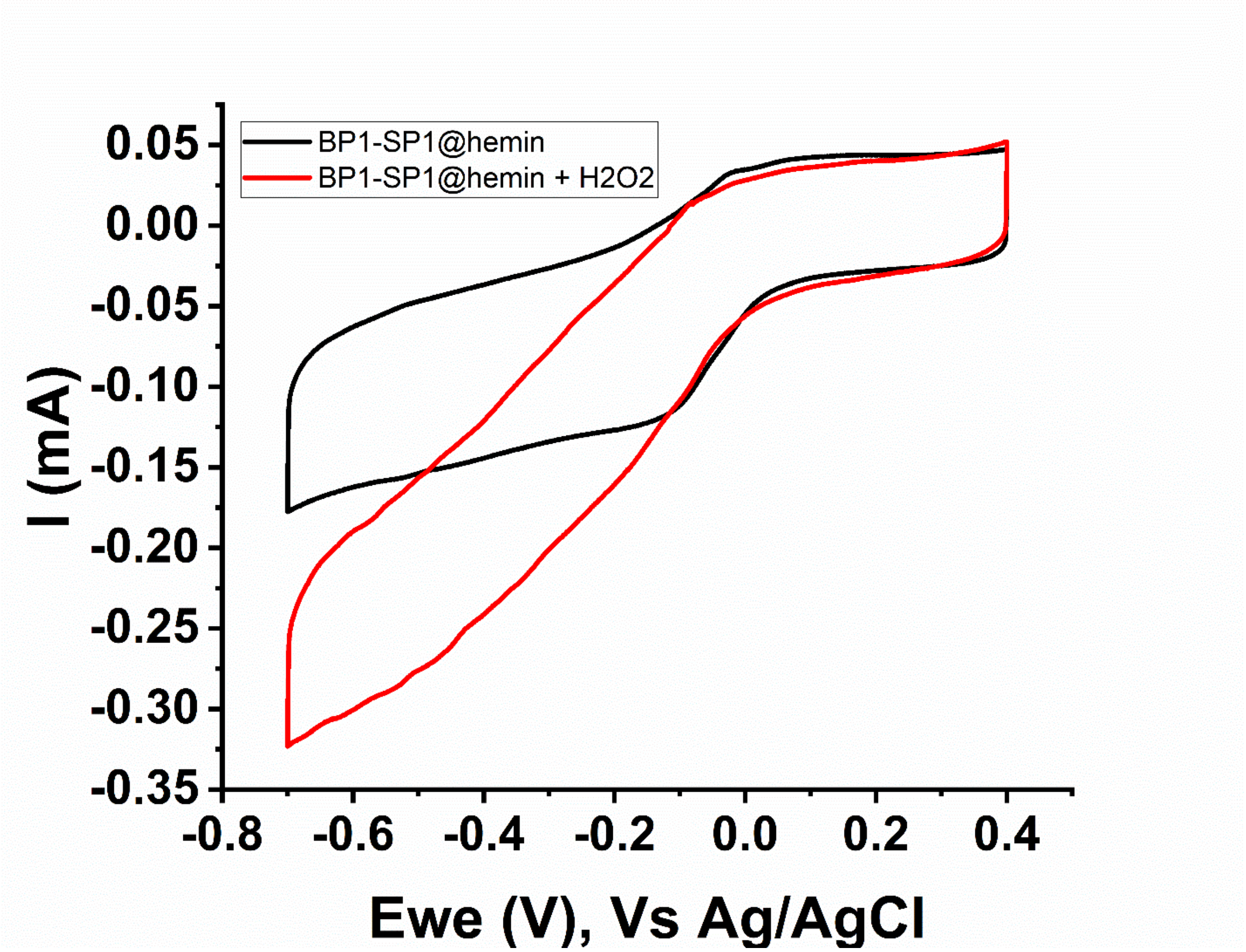

Figure S5. Cyclic voltammogram of BP1-SP1@hemin in 95% 10mM HEPES and 5% DMSO at glassy carbon electrode with and without H_2O_2. The measurement was performed in PB 0.1 M, pH = 7.5 at 5 mV/s in anaerobic conditions.

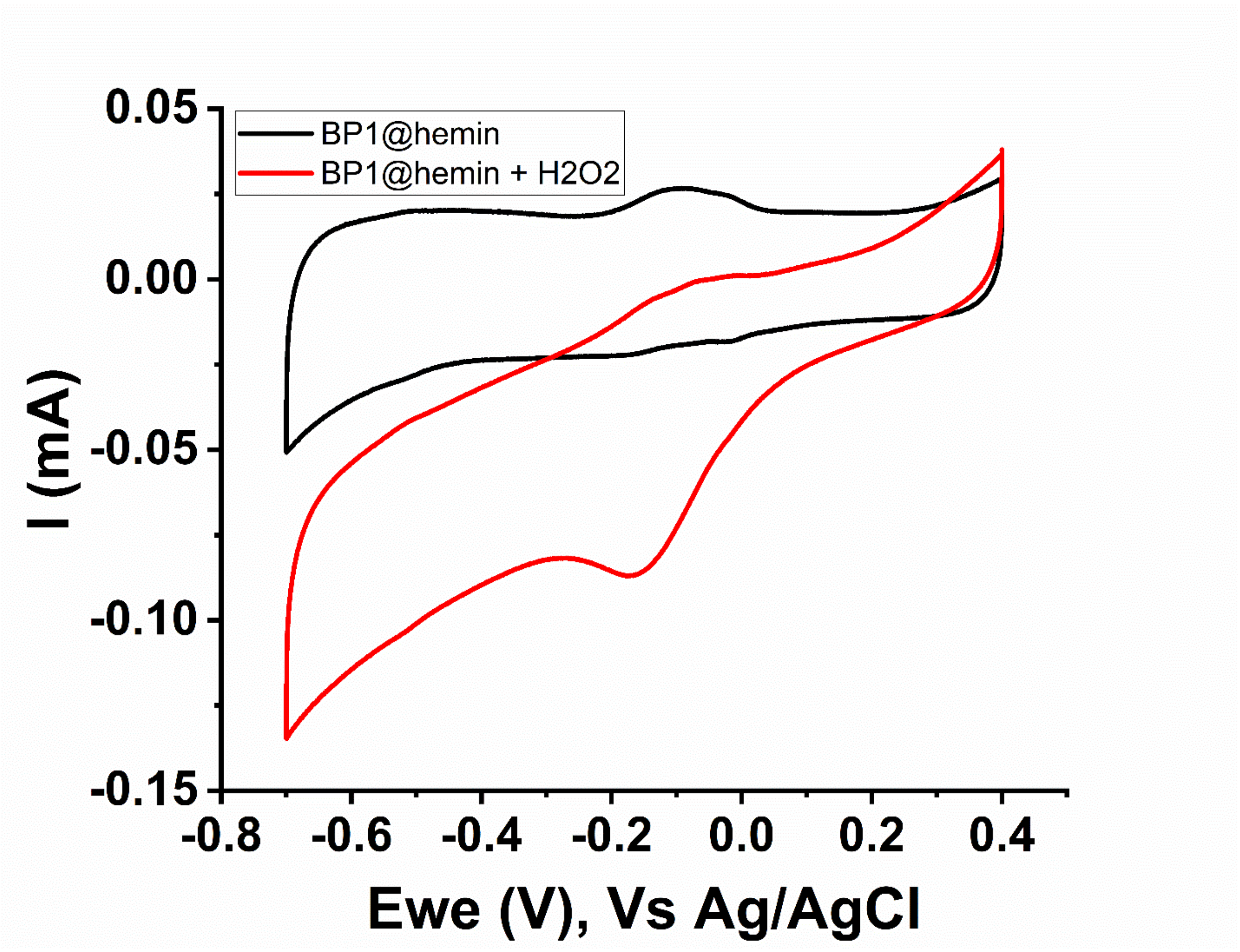

Figure S6. Cyclic voltammogram of BP1@hemin in 95% 10mM HEPES and 5% DMSO at glassy carbon electrode with and without H_2O_2. The measurement was performed in PB 0.1 M, pH = 7.5 at 5 mV/s in anaerobic conditions.

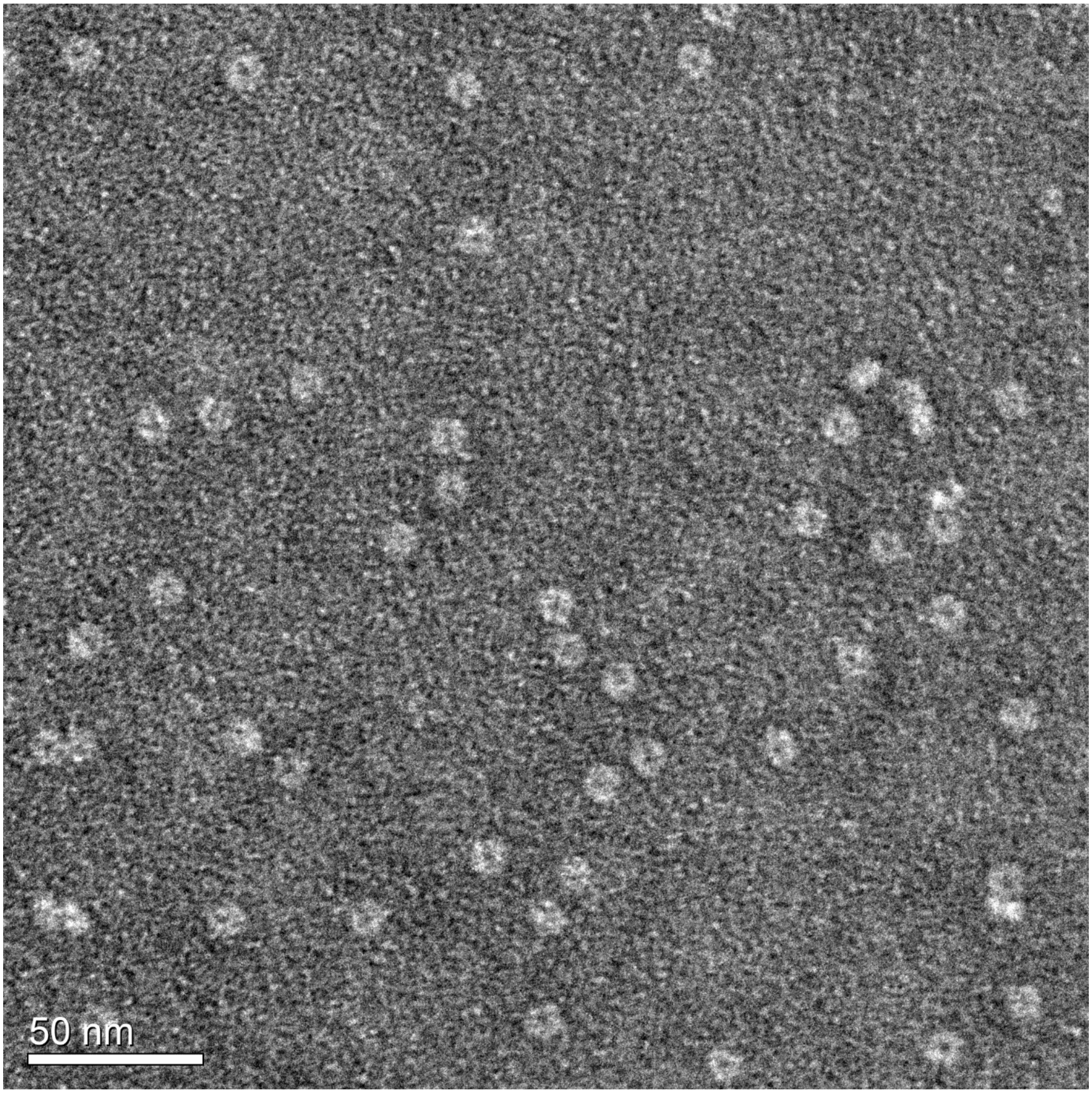

Figure S7. transmission electron microscopy image of BP1-SP1 dissolved in 95% 10mM HEPES and 5% DMSO, negatively stained by uranyl acetate 1%

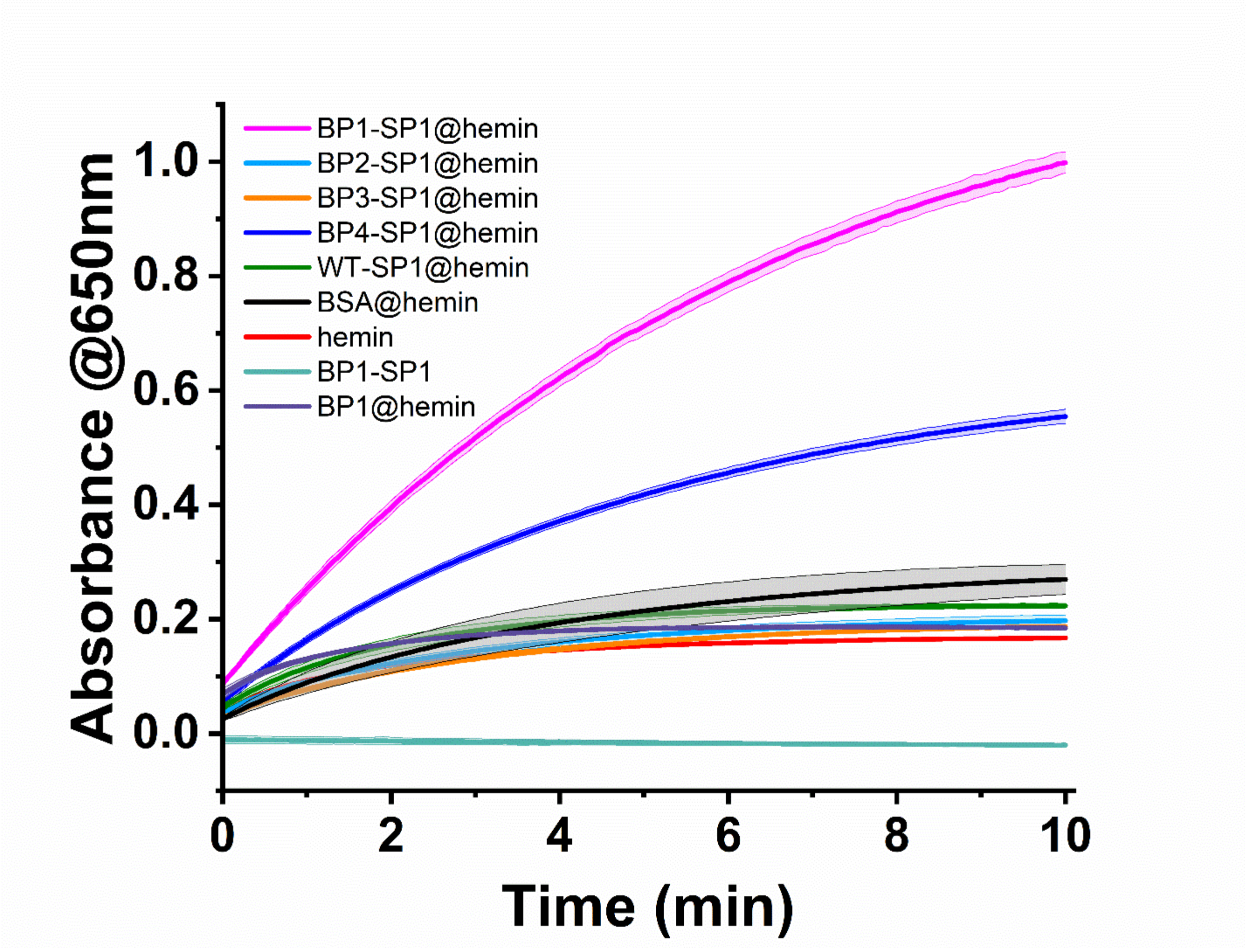

Figure S8. peroxidase-like activity of different SP1 variants with hemin in the presence of H_2O_2 1.28mM, ABTS 1.28mM, BP1-SP1 0.25μM, hemin 2μM, HEPES buffer 10mM pH=8, the reaction was initiated by the addition of H_2O_2. The absorbance of ABTS was measured at 650nm for 10 minutes. The light area around the curve indicates the standard deviation of three independent experiments.

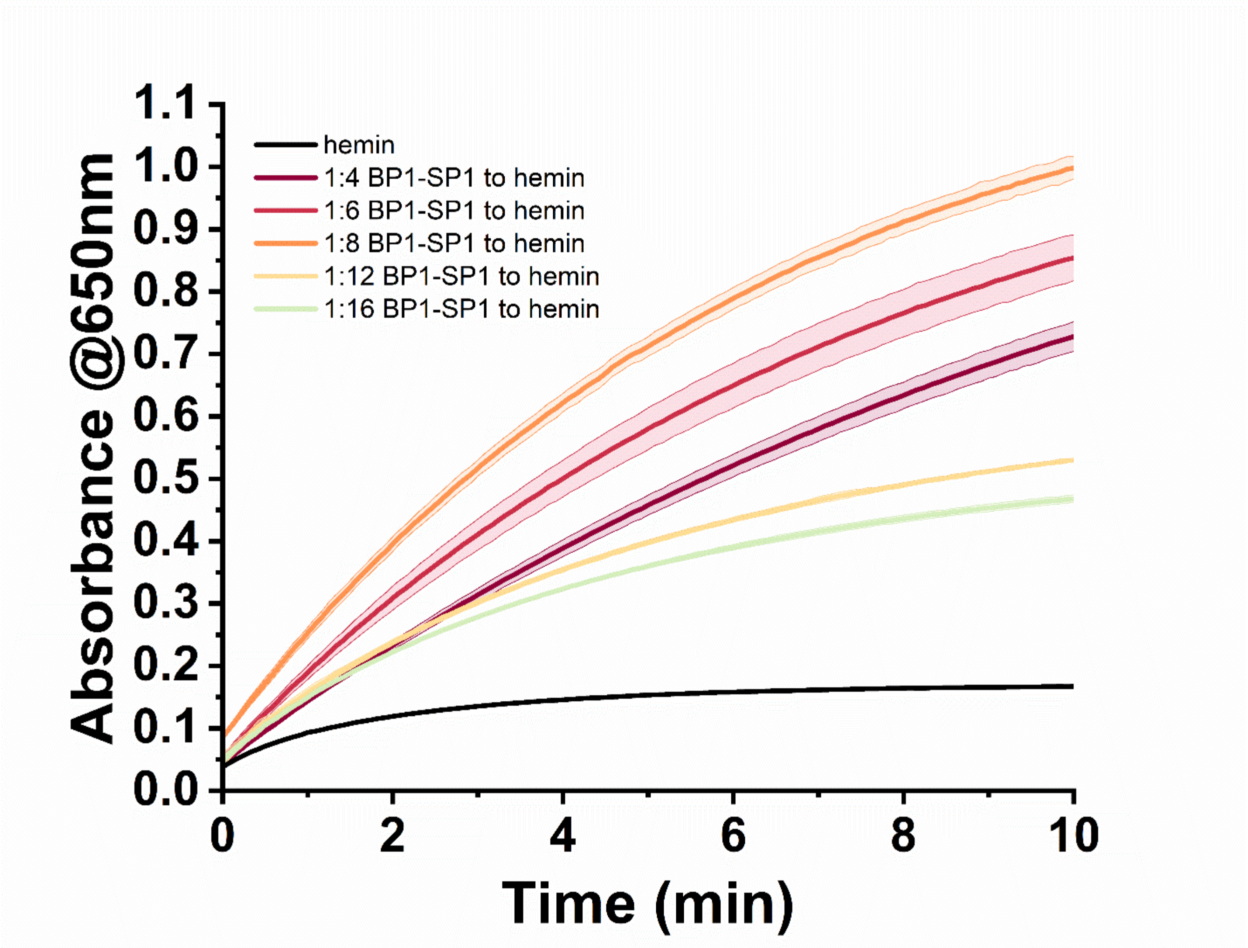

Figure S9. peroxidase-like activity of the BP1-SP1@hemin complex at different protein to hemin ratios in the presence of H_2O_2 1.28mM, ABTS 1.28mM, BP1-SP1 concentration was varied from µM, hemin 2µM, HEPES buffer 10mM pH=8, the reaction was initiated by the addition of H_2O_2. The absorbance of ABTS was measured at 650nm for 10 minutes. The light area around the curve indicates the standard deviation of three independent experiments.

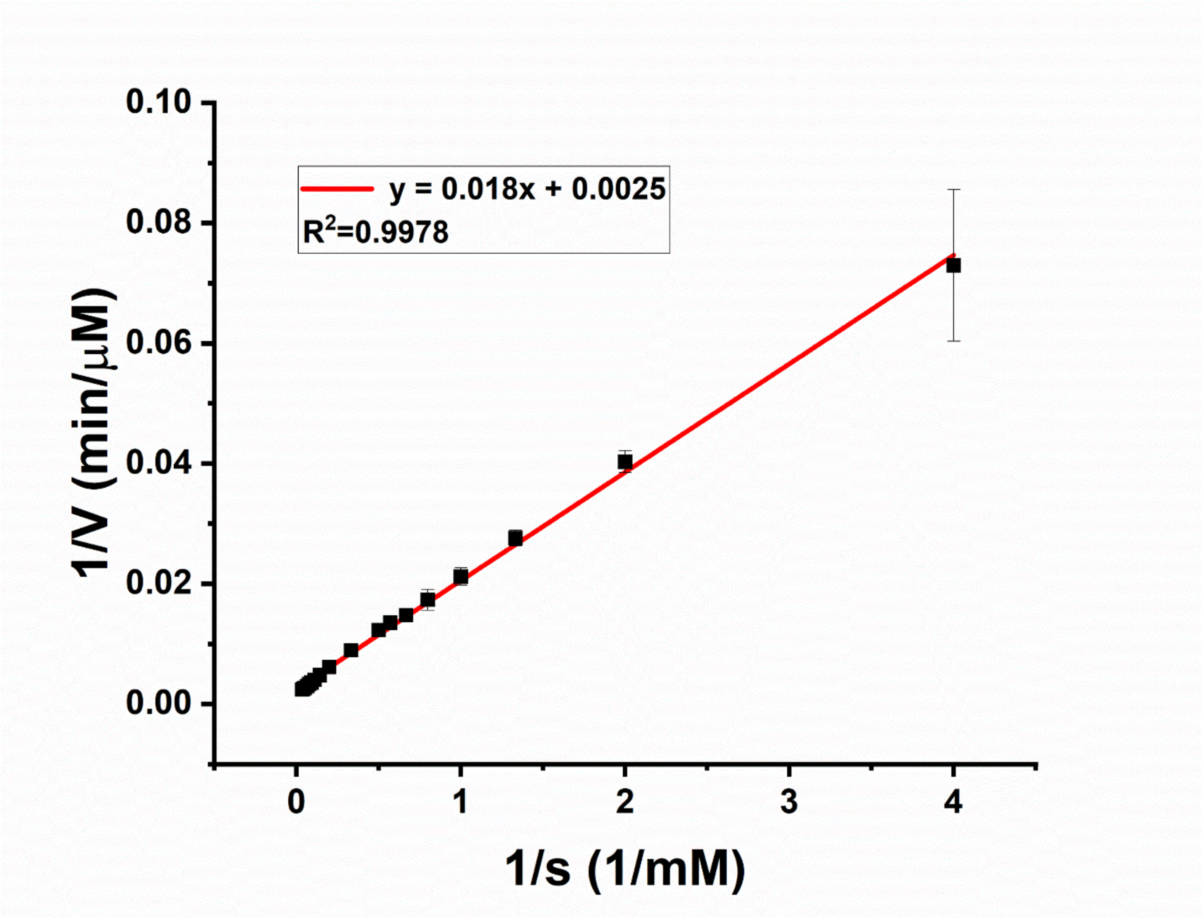

Figure S10. Lineweaver-Burk analysis of BP1-SP1@hemin complex. 2.3µM of hemin and 0.29µM of SP1, 10mM ABTS, and the different concentrations of H_2O_2. The reaction was initiated by the addition of H_2O_2.

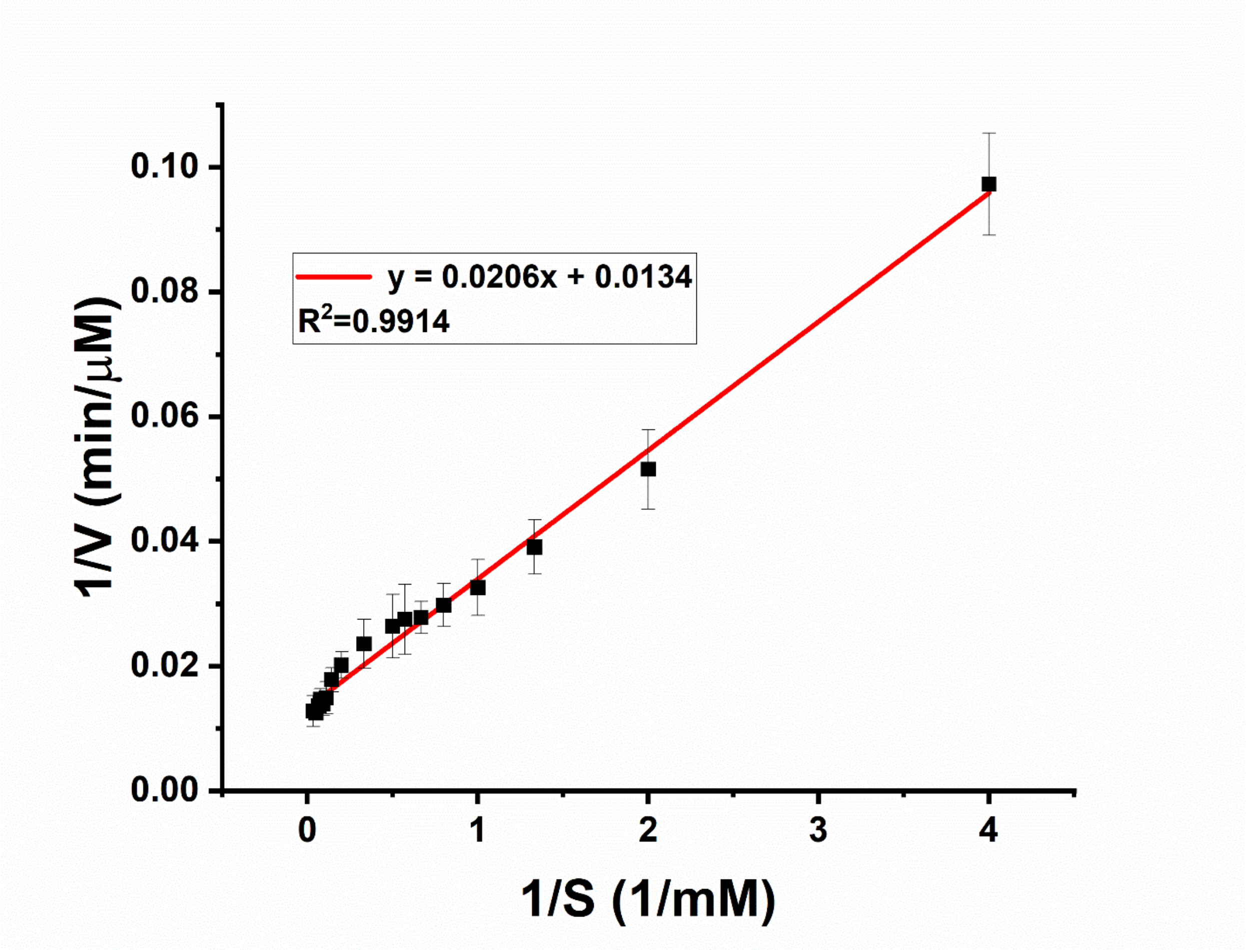

Figure S11. Lineweaver-Burk analysis of hemin 2.3μM of hemin, 10mM ABTS, and the different concentrations of H_2O_2. The reaction was initiated by the addition of H_2O_2.

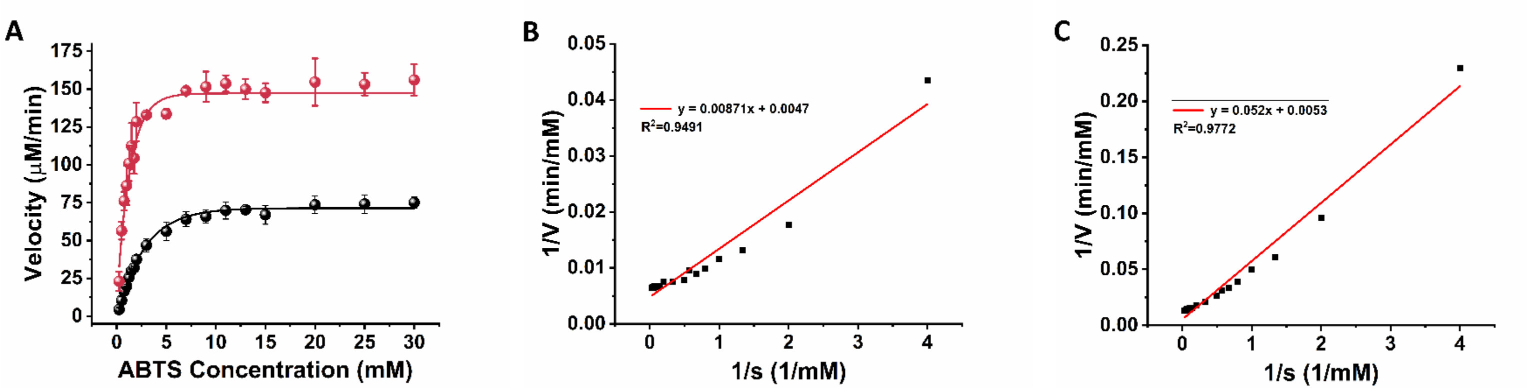

Figure S12. (A) The Michalis-Menten kinetic curve of BP1-SP1@hemin complex (red dots) and hemin (black dots), H_2O_2 10mM, BP1-SP1 0.29µM, hemin 2.3µM, HEPES buffer 10mM pH=8 and the different concentrations of ABTS. The reaction was initiated by the addition of H_2O_2. The absorbance of ABTS was measured at 650nm for 1 minute. Error bars indicate the standard deviation of independent triplicates. Lineweaver-Burk analysis of (B) BP1-SP1@hemin and (C) hemin.

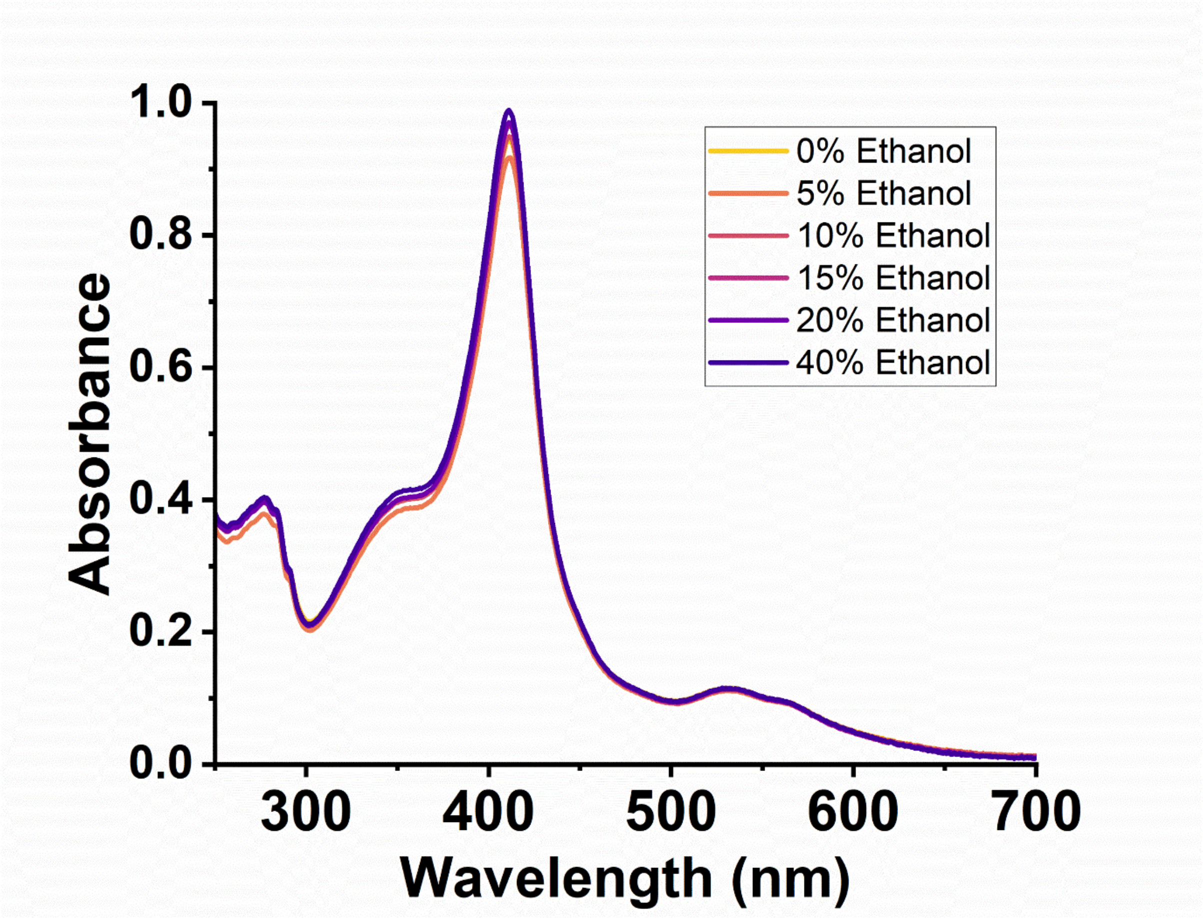

Figure S13. Absorption spectra of BP1-SP1@hemin complex (BP1-SP1 2.5µM and hemin 20µM) in the presence of at different ethanol concentrations.

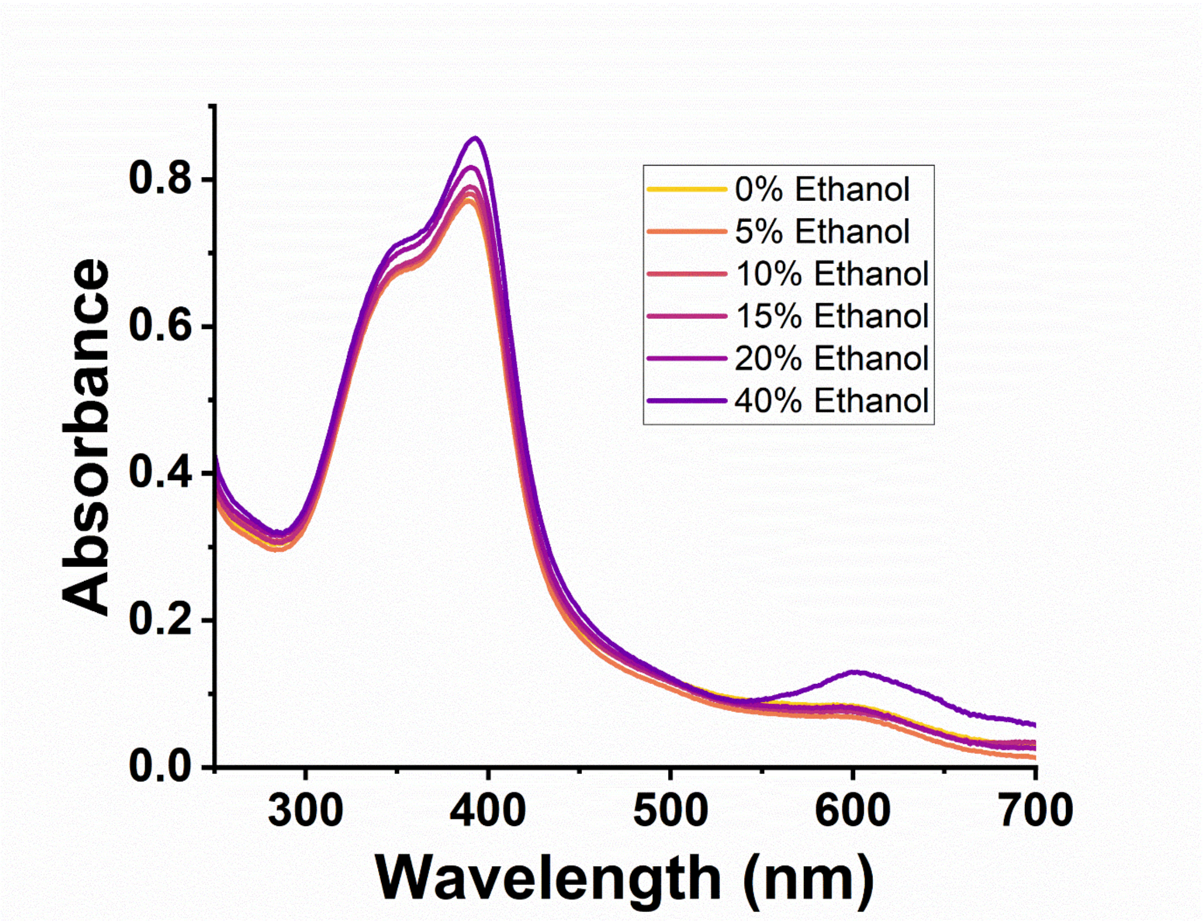

Figure S14. Absorption spectra of 20µM hemin in the presence of different ethanol concentrations

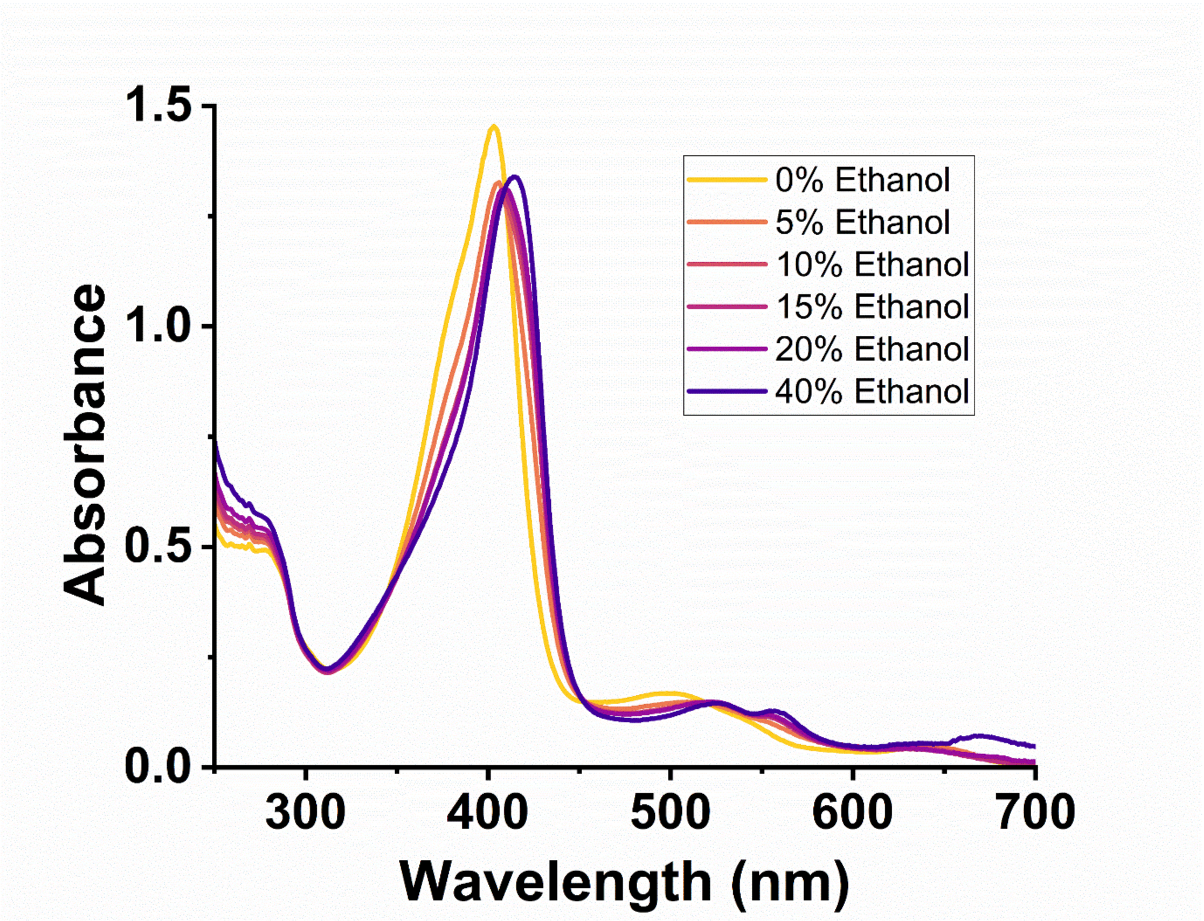

Figure S15. Absorption spectra of 1mg/ml HRP in the presence of different ethanol concentrations

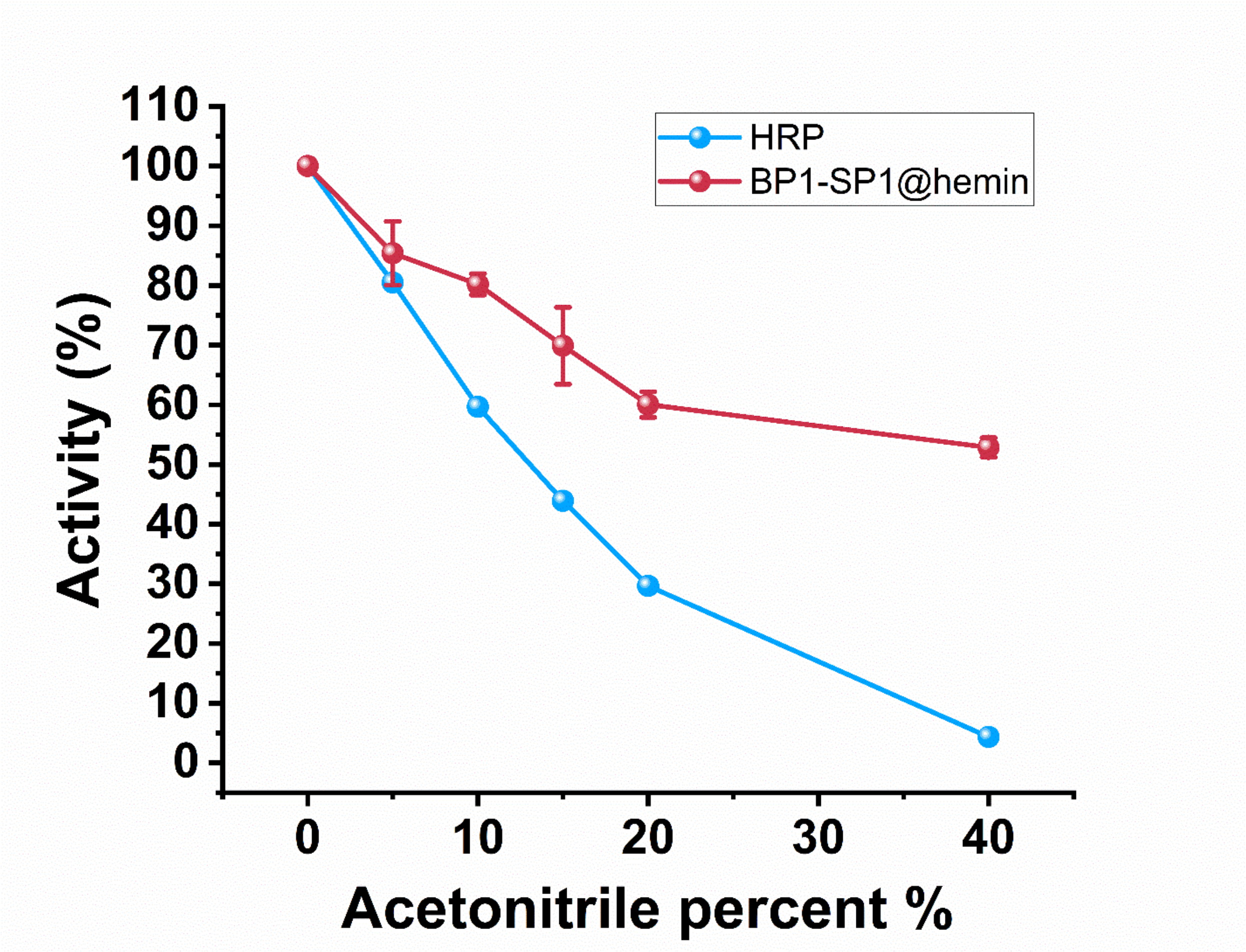

Figure S16. Relative activity of BP1-SP1@hemin complex (red), free hemin (black), and HRP (blue) in different acetonitrile percentages, H2O2 1.28mM, ABTS 1.28mM, BP1-SP1 0.25µM, hemin 2µM, HEPES buffer 10mM pH=8. The reaction was initiated by the addition of H2O2. The absorbance of ABTS was measured at 650nm for 10 minutes. Error bars resemble the standard deviation of independent triplicates.

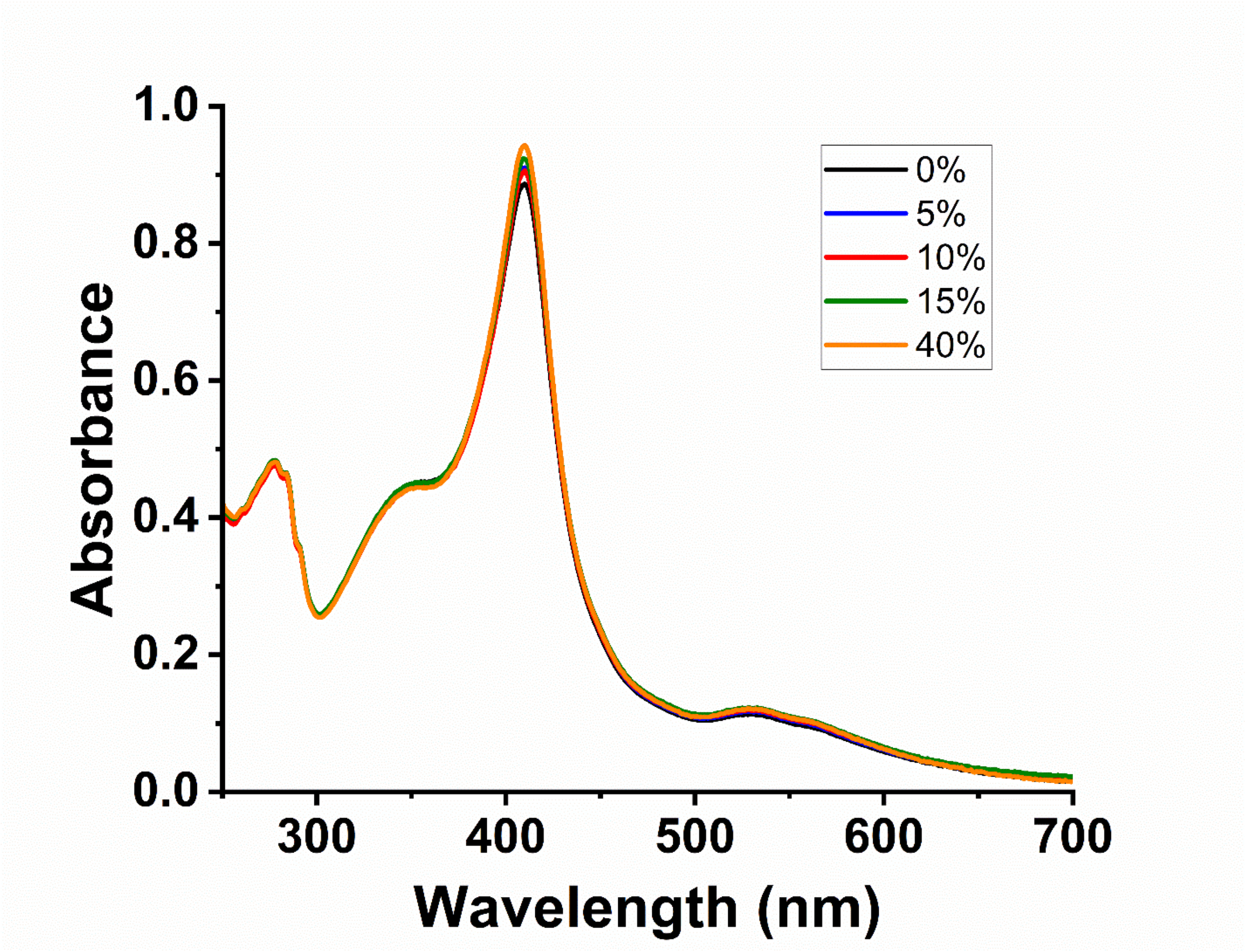

Figure S17. Absorption spectra of BP1-SP1@hemin complex (BP1-SP1 2.5µM and hemin 20µM) in the presence of at different acetonitrile concentrations.

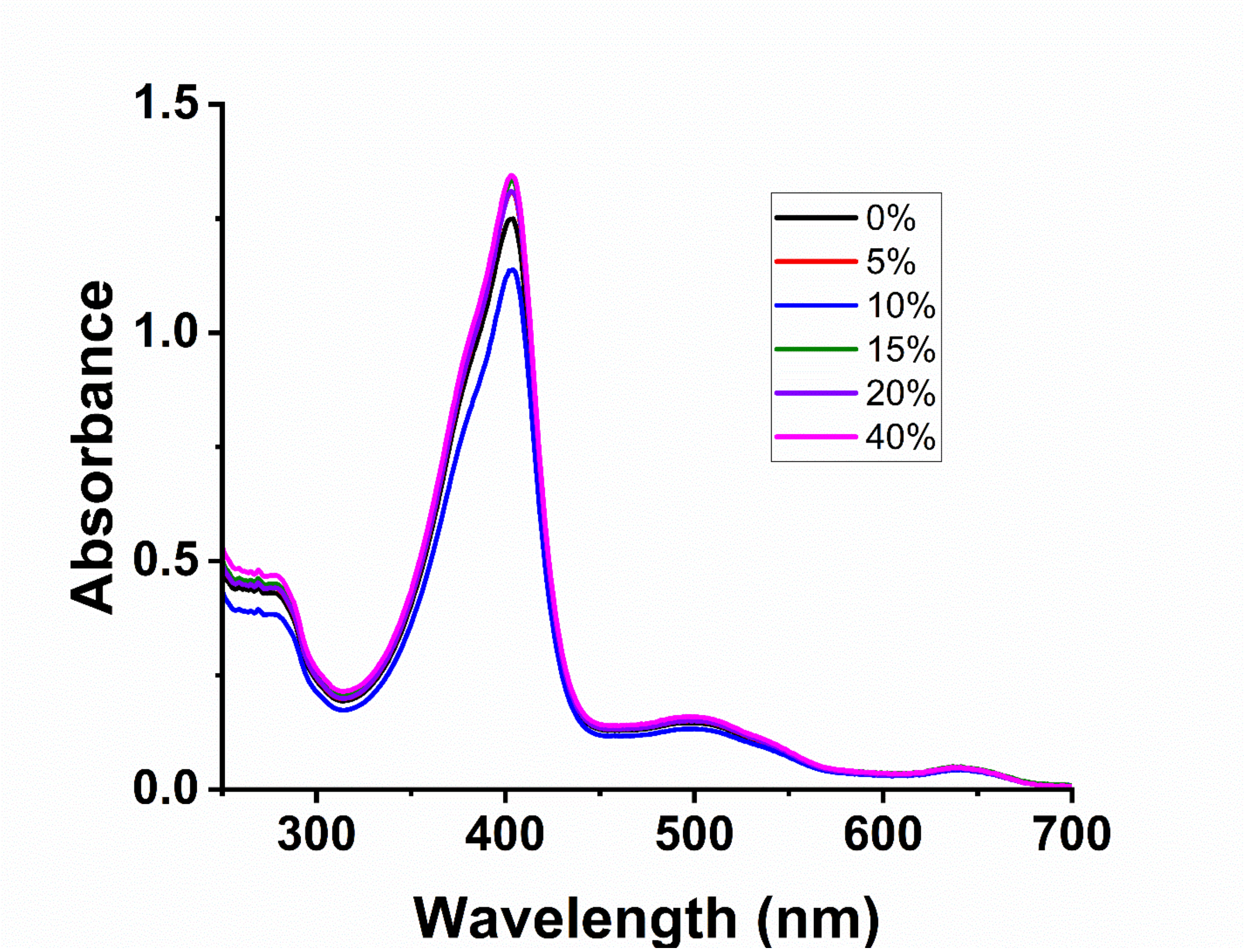

Figure S18. Absorption spectra of 1mg/ml HRP in the presence of different acetonitrile concentrations

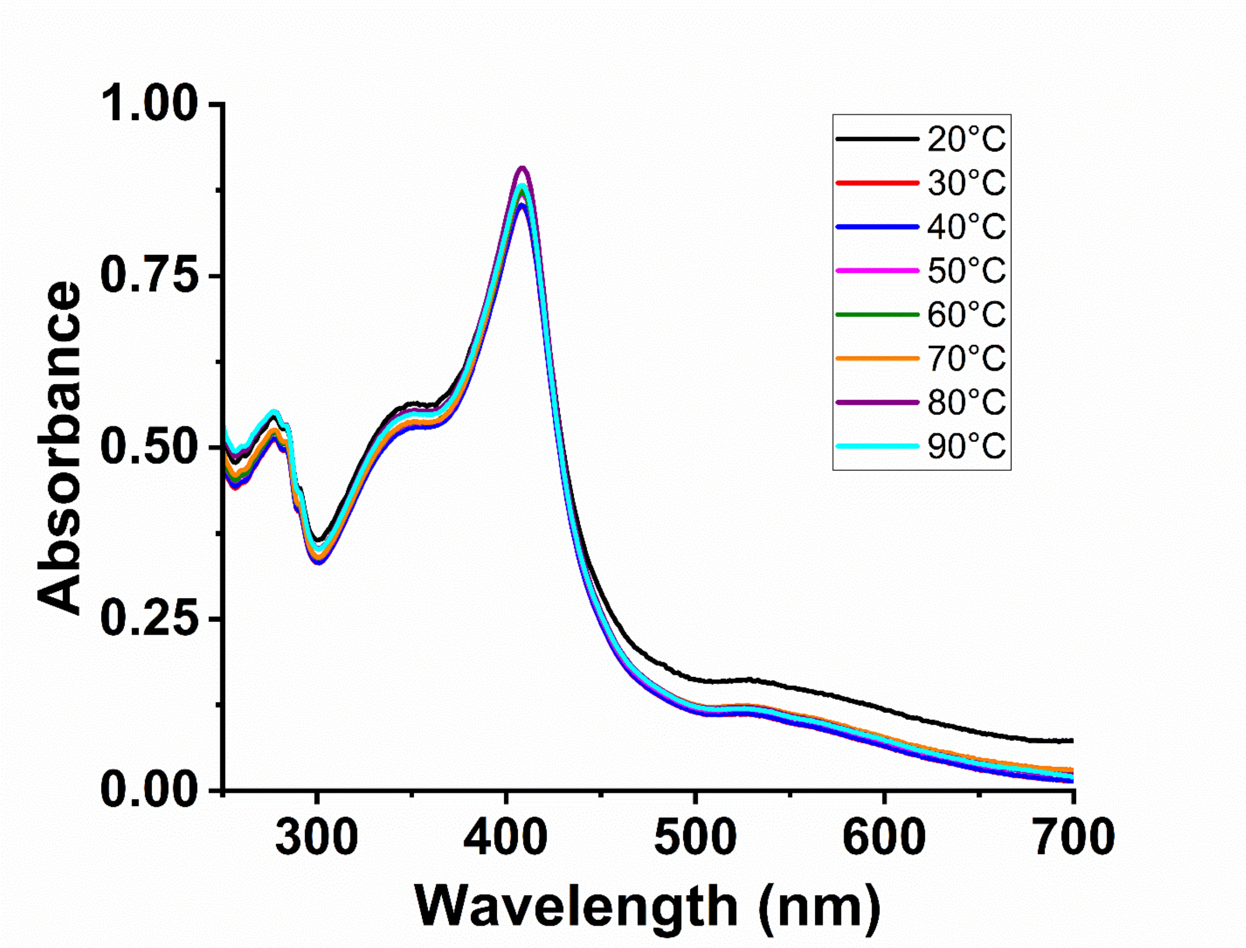

Figure S19. Absorption spectra of BP1-SP1@hemin (BP1-SP1 2.5µM and hemin 20µM) at different temperatures. The biohybrids and hemin solution consists of 95% HEPES and 5% DMSO

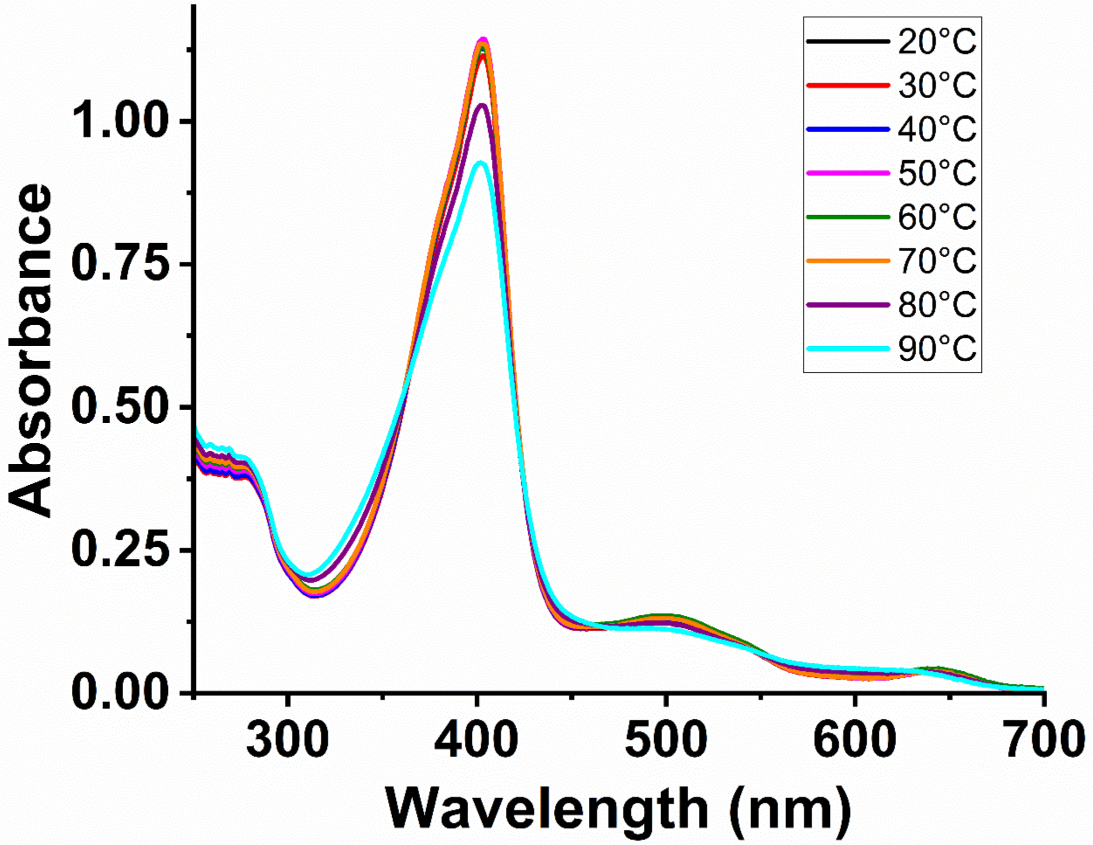

Figure S20. Absorption spectra of 1mg/ml HRP dissolved in 95% HEPES and 5% DMSO at different temperatures

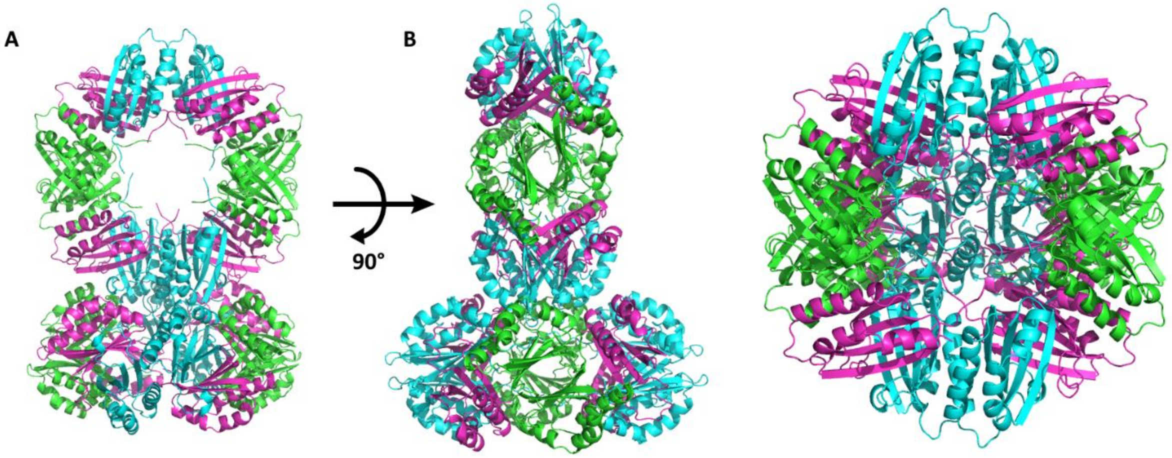

Figure S21. Crystal structure of WT SP1. (A) Ribbon representation of two dodecameric rings formed by trimers in different asymmetric units. (B) View from the bottom of the ring showing the tight interaction across the rings.

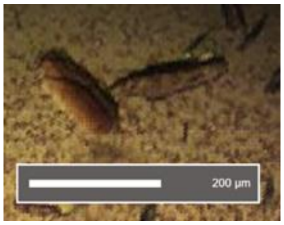

Figure S22. BP1-SP1@hemin crystal.

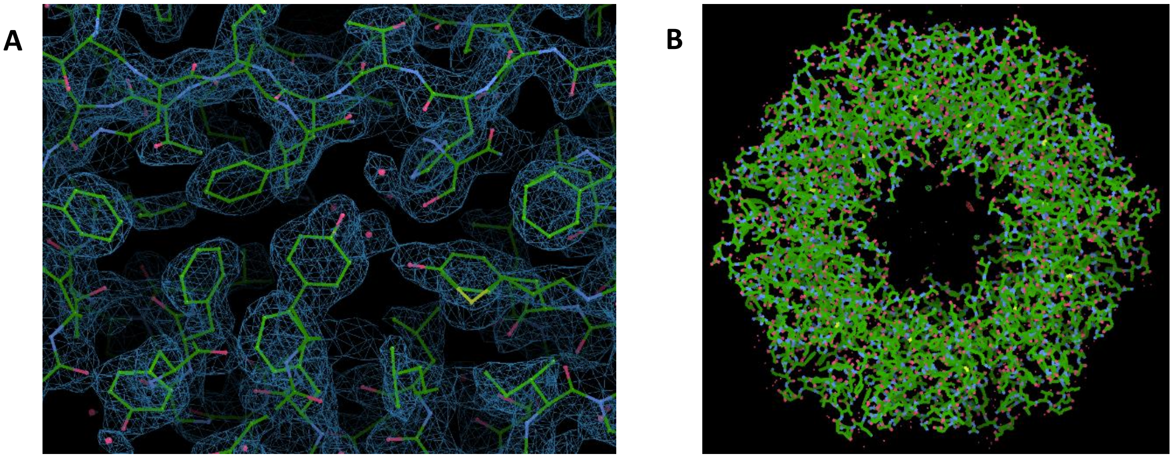

Figure S23. Electron density map of BP1-SP1@hemin crystal structure. A) 2Fo-Fc map (contoured at 1σ), showing the quality of the data. B) Fo-Fc map showing the empty internal cavity of the structure (contoured at 3σ).

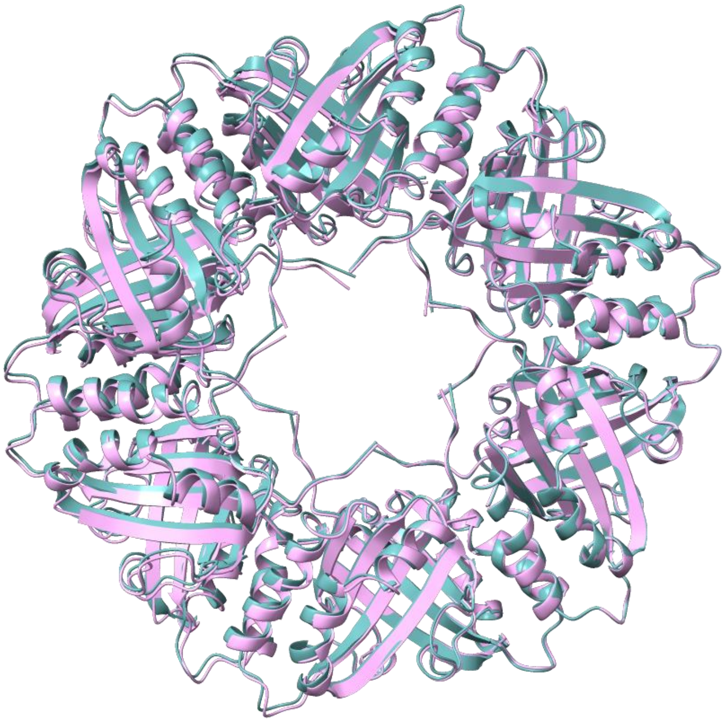

Figure S24. Superposition of single dodecameric rings of the WT-SP1 and BP1-SP1@hemin complex. The rmsd over all C_α atoms is 0.278Å

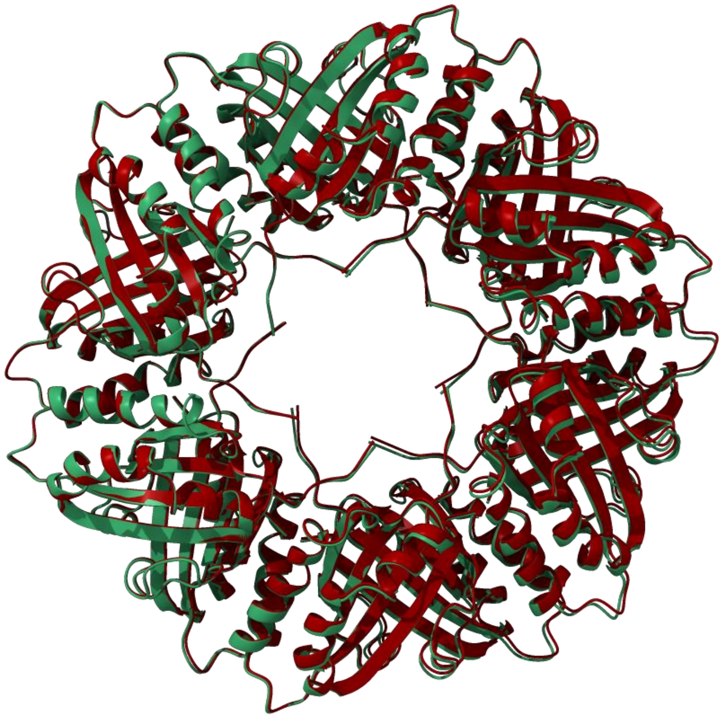

Figure S25. Superposition of single dodecameric rings of the BP1-SP1 and BP1-SP1@hemin complex. The rmsd over all C_{α} atoms is 0.188Å

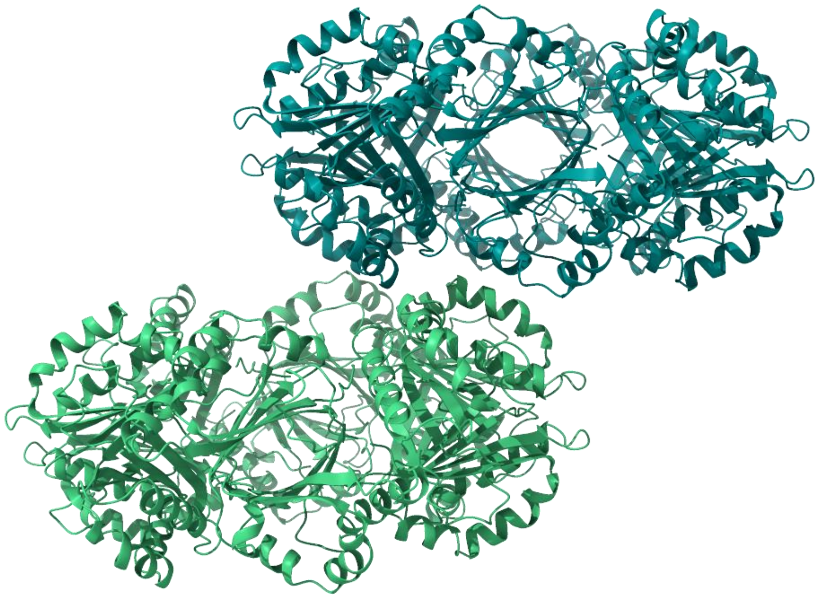

Figure S26. Asymmetric unit of the BP1-SP1
